# Visual–Inertial Fusion Framework for Isolating Seated Human-Body Vibration in Dynamic Vehicular Environments

**DOI:** 10.3390/s26041355

**Published:** 2026-02-20

**Authors:** Nova Eka Budiyanta, Azizur Rahman, Chi-Tsun Cheng, George Wu, Toh Yen Pang

**Affiliations:** 1 Biomedical Engineering Department, School of Engineering, STEM College, RMIT University, Melbourne, VIC 3000, Australia; nova.eka@atmajaya.ac.id; 2 Department of Electrical Engineering, School of Bioscience, Technology, and Innovation, Universitas Katolik Indonesia Atma Jaya, BSD City 15345, Banten, Indonesia; 3 Atma Jaya AI Research Center, Universitas Katolik Indonesia Atma Jaya, Jakarta 12930, Indonesia; 4 Construction Management Department, School of Property, Construction and Project Management, College of DSC, RMIT University, Melbourne, VIC 3000, Australia; azizur.rahman@rmit.edu.au; 5 Mechanical, Manufacturing and Mechatronic Engineering Department, School of Engineering, STEM College, RMIT University, Melbourne, VIC 3000, Australia; ben.cheng@rmit.edu.au (C.-T.C.); george.wu@rmit.edu.au (G.W.)

**Keywords:** whole-body vibration, visual–inertial sensor fusion, in-vehicle human posture dynamic

## Abstract

Understanding how seat-induced whole-body vibration (WBV) is transmitted to and actively compensated by the human body is essential for accurately assessing discomfort, fatigue, and postural control in vehicle occupants. This study proposes a visual–inertial fusion framework utilizing IMU-RGB-D data to isolate seated human body vibration in dynamic vehicular environments. In real-cabin monitoring systems, measured motion is a superposition of platform vibration, passive transmission through the body, active postural compensation, and camera jitter. Existing WBV and driver monitoring studies typically rely on single modality sensing, such as inertial or visual approaches, without decomposing these components or modelling camera vibration. The framework synchronized three IMUs with RGB-D landmarks. Seat, human body, and camera accelerations are separated, and body vibration velocity is derived from body–seat differential acceleration via band-pass filtering and spectral integration. The 3D landmarks enable rotational-translational Postural Compensation Index metrics, axis-wise energy distributions, and anthropometric consistency checks. The study is held in an in-service urban tram case. Torso vibration is dominated by 40% anteroposterior components, while head postural is predominantly > 50% lateral sway. Near static anthropometric evaluation was also studied, resulting in shoulder width errors that remain within ±10–20 mm. The results show that the framework can distinguish passive ride phases from strongly compensated phases, separate camera jitter from true body motion, and reveal anisotropic postural strategies, providing a structured basis for vibration and posture analysis in in-vehicle monitoring.

## 1. Introduction

Modern transport systems rely on millions of professional drivers who spend long hours sitting in moving vehicles, so even modest decrements in comfort, health, or alertness can accumulate into substantial safety risks and economic costs for the transport sector. It happens because seated occupants in cabin vehicles such as cars, buses, forklifts, trucks, and rail vehicle are continuously exposed to whole-body vibration (WBV) generated by suspension dynamics, road or rail irregularities, powertrain operation, and inertial forces during acceleration and braking [1,2,3], which motivates the need for physically grounded measurement and decomposition of in-vehicle motion for subsequent vibration and postural response analysis. This excitation is transmitted through the vehicle structure to the human body through the seat. Even when the overall driving posture appears static, the musculoskeletal system is subjected to continuous low- to mid-frequency vibration [2,3,4]. For drivers, prolonged exposure to such WBV is associated with discomfort, increased neuromuscular effort to maintain posture and fatigue, which in turn can degrade alertness and vehicle control [5,6,7]. For applications in ergonomics and driver monitoring, it is therefore important not only to quantify seat vibration, but also to understand how much vibration is experienced by the body and how strongly the body responds through postural compensation.

Sensing technologies for human monitoring in vehicles have advanced in two main streams. First, inertial measurement units (IMUs) mounted on the seat and the body provide direct measurements of linear acceleration and angular velocity [8,9]. IMUs capture how vibration is transmitted and how the torso moves. Second, RGB-D cameras and model-based pose estimators are used for contactless 3D skeletal tracking of key landmarks such as the head and shoulders [10,11]. However, each stream suffers from a fundamental ambiguity in dynamic vehicular environments. A body-attached IMU measures the superposition of vibration induced by the seat, mechanically transmitted motion through the body, and active postural compensation [12,13]. An in-vehicle RGB-D camera is rigidly attached to the vibrating vehicle structure, so the same structural vibration that reaches the seat also reaches the camera, causing camera jitter [14]. This jitter induces apparent motion of all 3D landmarks even when the body segment is momentarily still. In practice, most vision-based in-vehicle monitoring implicitly assumes a static or perfectly stabilized camera, while inertial measurements alone cannot disentangle seat-driven motion from voluntary body movement. Therefore, the primary objective of this study is to develop and demonstrate a multimodal IMU-RGB-D sensing and analysis framework that explicitly separates seat excitation, body response, and camera motion, rather than to directly infer comfort, fatigue, or other human-factor outcomes.

As a result, neither stream can reliably isolate true seated human body vibration from platform-induced artefacts. For instance, signals from IMU sensors are difficult to determine whether a change in orientation or acceleration arises from seat motion, passive transmission, or active postural control [15,16]. Readings from RGB-D landmarks are difficult to determine whether a change in head or shoulder position reflects actual postural adjustments or simply camera motion relative to the reference. The error is compounded by the fact that vehicle vibration and human postural responses occupy overlapping frequency bands, typically in the range of about 1–20 Hz [17,18,19]. Simple filtering is not sufficient to separate platform vibration from postural compensation when both lie in the same spectral region, and visual stabilization methods that ignore sensor dynamics cannot fully handle structural jitter of the camera mount.

Existing work on in-vehicle vibration and posture typically treats seat vibration, body vibration, and postural responses in isolation. Classical WBV studies focus on seat vibration and transmissibility from seat to body, often model the human as a passive mechanical system, and assume static or well-controlled sensor geometries [20,21]. Biomechanics and postural stability studies examine human responses to mechanical perturbations, but are usually conducted in controlled laboratory environments with stationary cameras and well-characterized support surfaces [22]. Driver monitoring and RGB-D-based skeleton tracking studies mainly assume that the visual sensor is static or apply general-purpose video stabilization, without explicitly modelling camera jitter as a vibration layer [23]. Therefore, there is limited exploration of the joint structure of seat IMU, body IMU, camera IMU and RGB-D landmarks to decompose observed motion into meaningful components.

Most existing in-vehicle studies rely on a single sensing stream, such as IMU-based vibration signals or vision-based skeleton tracking, and often implicitly assume a static camera or treat camera motion as measurement noise. In contrast, we combine seat, body, and camera IMUs with RGB-D landmarks in a unified formulation to explicitly separate seat excitation, body response, and camera-induced jitter affecting the 3D landmarks. This fusion is used not only for redundancy, but as a physically grounded decomposition front-end that reduces platform and camera-induced artefacts before interpreting postural motion and computing transmission and compensation metrics.

The core problem addressed in this work is to develop a framework that can isolate seated human body vibration and postural compensation from platform vibration and camera jitter, using realistic in-vehicle sensing. Conceptually, the measured motion of a seated person inside a vibrating vehicle can be viewed as a superposition of four interacting components: 1. structural vibration transmitted from the vehicle to the seat, 2. mechanical vibration transmitted through the body due to contact with the seat, 3. active postural adjustments generated by neuromuscular systems in response to perturbation, and 4. camera jitter, which maps structural vibration into apparent skeleton motion in RGB-D data [4,16,20]. All four components are present simultaneously and interact in time, frequency and space. Without an explicit way to decompose them, interpretations of IMU and RGB-D data remain ambiguous, limiting progress in ergonomic assessment related to vibration in pre-fatigue studies and human monitoring systems in real vehicles. This leads to the following research questions (RQ):

- RQ1—How can seat, body, and camera IMU signals be fused to decouple camera jitter and platform excitation from true body motion in realistic in-vehicle environments?
- RQ2—How can fused IMU-RGB-D measurements be used to distinguish passive mechanically transmitted vibration from active postural compensation at both rotational and translational levels?

To address these research questions, a key methodological gap is the absence of an integration IMU-RGB-D fusion framework that models vehicle seat excitation, quantifies seat vibration transmission to body vibration, isolates active postural compensation at both rotational and translational levels, and compensates camera jitters in RGB-D landmarks, all within a unified formulation suitable for dynamic environments. Accordingly, the present work is positioned as a proof-of-concept methodological framework, rather than a clinical validation, large-scale experimental study, or population-level assessment. Existing methods generally use one or two sensors, make implicit assumptions about static platforms or cameras, or treat platform vibration and camera jitter as noise rather than as structured signals to be modelled [10,12,15]. There is a need for a sensor fusion approach that treats seat vibration, body vibration, postural compensation and camera jitter as distinct but coupled layers, and that yields metrics directly tied to these components.

Accordingly, the aim of this study is to develop and demonstrate a visual–inertial fusion framework that can (i) separate seat, body, and camera vibration in a moving vehicle, (ii) distinguish passively transmitted vibration from actively compensated postural motion, and (iii) quantify the stability of RGB-D skeletal landmarks under realistic cabin vibration. This aim is pursued by implementing an IMU-RGB-D fusion framework in an in-service urban tram. All channels are synchronized on a common time base and combined into a unified data matrix after frame-level quality masking. On the inertial side, seat to body and seat to camera vibration transmission are quantified in the time domain using sliding window root mean square (RMS) and the resulting vibration transfer ratio (VTR). In addition, a body vibration velocity metric is derived from differential acceleration between body and seat, after bandpass filtering and spectral integration, to better reflect vibration intensity in terms of comfort and fatigue for further study. On the visual side, camera orientation is estimated from the camera IMU quaternion and used to perform reverse orientation of 3D landmarks reconstructed from the RGB-D stream. These motion-compensated landmarks are mapped into a world coordinate frame using extrinsic parameters obtained from checkerboard calibration.

To evaluate the proposed visual–inertial fusion framework, the Postural Compensation Index (PCI) is introduced in this study, besides VTR, to indicate how active the body is in stabilizing itself under vibration, including rotational postural compensation and translational postural compensation. Rotational postural compensation is quantified by a rotational PCI, defined as the sliding RMS of the difference between body and seat angular velocities. In addition, translational postural compensation is quantified by a translational PCI, defined as the sliding RMS of pseudo velocity in the world frame of each landmark. This yields segment-specific indices for the nose representing the head and shoulders, capturing how different upper body segments contribute to postural adjustments under vibration. Axis-wise analysis of both differential body acceleration and landmark pseudo velocity reveals anisotropic control strategies across lateral (x), vertical (y), and anteroposterior (z) directions. Finally, anthropometric consistency metrics based on 3D shoulder width and nose–mid-shoulder distance are used to assess the geometric stability of the fused skeleton under vibration and jitter.

Taken together, the proposed framework treats seat vibration, body vibration, postural compensation, and camera motion as distinct but coupled components within a single visual–inertial pipeline. The following sections describe its implementation in an in-service tram and evaluate how the derived metrics characterize seat-to-body vibration transmission, postural compensation patterns, and RGB-D skeleton stability in a realistic transport environment. Section 2 reviews related works. Section 3 provides materials and methods proposed in this study, as well as their related works. Section 4 presents experimental results from the tram case study and interprets how the vibration and PCI metrics interact. Section 5 discusses implications, limitations, and possible extensions of the framework, and Section 6 concludes the paper.

## 2. Related Work

### 2.1. Whole-Body Vibration and Seated Biodynamic Response

WBV refers to mechanical oscillations that are transmitted to the human body as a whole, typically through the supporting surfaces of a seat, floor, or backrest in vehicles and workstations [16,19]. In seated occupants of vehicles, vibration is conveyed from the vehicle structure to the seat pan and back rest and then through the pelvis and spine into the upper body and head [16,24]. Even when the overall posture appears static, the musculoskeletal system is subjected to continuous low to mid-frequency excitation, which can affect comfort, fatigue, and long-term health [5,19].

The biodynamic response of the seated human to such excitation has classically been modelled using viscoelastic mechanical analogues composed of mass, spring, and damper elements. In single degree of freedom (1 DOF) models, the body is represented by a lumped mass supported by a vertical spring-damper system, capturing the dominant resonance in the pelvis-spine direction under vertical seat vibration [25,26]. More detailed multi-DOF models subdivide the body into body segments interconnected by viscoelastic elements, allowing separate characterization of resonances in lateral, vertical, and anteroposterior directions [23,26]. These models provide a compact description of how externally applied seat acceleration is filtered and amplified as it propagates through the body.

Australian Standard 2670 adopts ISO 2631 for the evaluation of human exposure to WBV and ISO 5982 for the biodynamic response of the seated person formalize several of these concepts. They specify frequency-weighting curves, exposure durations, and recommended limits for comfort and health, as well as reference biodynamic response functions that a mechanical model or seat design should approximate [19,27]. Within this framework, vibration is typically characterized in terms of acceleration root mean squared (RMS), weighted frequency and response functions describing transmissibility from the seat input to body segments. In many studies, seat–body transmissibility is analyzed in the frequency domain as the ratio of body acceleration spectrum to seat acceleration spectrum, highlighting resonant peaks and attenuation regions [20,28]. In some studies, vibration velocity or derived comfort indices have also been considered, as velocity-based measures can show stronger correlations with perceived comfort and fatigue than unweighted acceleration alone [19,29,30].

Most WBV studies, however, implicitly treat the seated human as a predominantly passive mechanical system driven by seat excitation. The focus is on how much of the seat vibration is transmitted to the body in different directions and frequency ranges, and how this transmission relates to comfort limits and health risks [20,24,26,31]. Active postural control and segment-specific adjustments of the upper body are often not modelled explicitly, but rather absorbed into effective stiffness and damping parameters or interpreted qualitatively [26,32,33]. Furthermore, the sensing configuration is usually limited to accelerometers or IMUs on the seat and selected body locations, with the seat regarded as the only relevant interface to the vehicle [34,35,36]. Visual sensing and potential motion of the camera or other sensors are not considered as separate vibration layers.

### 2.2. In-Vehicle Vibration and Seat–Body Transmissibility

A central concept in this literature is seat–body transmissibility, which describes how input vibration at the seat is amplified or attenuated by the human body. In many studies, transmissibility is estimated in the frequency domain as the ratio of the magnitude of the body acceleration spectrum to that of the seat, often focusing on vertical or anteroposterior directions [28,33]. These analyses reveal resonant frequency bands for the pelvis and lower spine, and higher resonances involving the thorax and head [28,37]. The shape of the transmissibility curve is then related to subjective comfort ratings, seat designs, suspension settings, and exposure limits [21,38]. In some cases, time-domain RMS measures are used in parallel to quantify overall vibration severity across driving scenarios such as highway cruising, urban traffic, or rough roads [39,40,41].

Research on in-vehicle vibration has also investigated the influence of seat properties, posture, and driving tasks. For professional drivers, long-term exposure has been linked to low back pain, musculoskeletal disorders, and fatigue [7,42,43,44]. Studies on trucks, buses, and off-road vehicles often compare different seat suspensions or isolation systems to reduce WBV at the driver’s seat [45,46]. In rail vehicles, including trams and trains, measurements along the track and at different vehicle speeds show how track irregularities, bogie dynamics, and vehicle design shape the vibration transmitted to passengers [47,48,49]. These works emphasize the importance of realistic operating conditions and long-duration measurements, moving beyond idealized laboratory excitations.

However, even in these more applied studies, humans are still largely treated as passive receivers of seat vibration. Transmissibility is typically computed between a seat sensor and a body sensor without explicitly modelling the distinction between mechanically transmitted vibration and active postural responses. Any active compensation is effectively folded into the observed transmissibility curves and sometimes discussed qualitatively, but not separated as a distinct signal component. Moreover, measurement configurations are usually limited to accelerometers on the seat and on a few body locations, and the analysis is expressed in terms of scalar acceleration metrics or single-axis transmissibility. Visual sensing, camera motion, and their interaction with vehicle and seat vibration are generally outside the scope of these works.

### 2.3. IMU-Based Monitoring of Seated Posture and Fatigue

A substantial line of work employs IMU features as indicators of fatigue or drowsiness. Changes in head nodding frequency, increased sway of the upper body, or alterations in movement smoothness have been associated with reduced alertness [50,51]. Machine-learning and pattern-recognition approaches have been applied to IMU time series to classify driver state into alert or drowsy and low or high fatigue [51,52]. In some studies, IMUs are combined with physiological signals, such as heart rate, or with vehicle-based measures, such as steering behaviour, to improve detection robustness [53,54]. These approaches treat IMU-derived kinematic features as convenient proxies for neuromuscular effort and postural stability under prolonged driving.

Multi-IMU setups have also been used to reconstruct seated upper-body kinematics with higher fidelity. By placing several IMUs along the spine and on the shoulders, researchers can infer joint angles and segment motions, sometimes using inverse kinematics or simplified biomechanical models [55,56]. Such systems have been deployed in driving simulators and laboratory mock-ups to examine how different seat designs, steering wheel positions, or tasks influence spinal loading and postural strategies [57,58]. In these contexts, the IMU network is primarily seen as a wearable motion-capture system that bypasses the need for optical markers and cameras.

However, in most of these IMU based monitoring studies, the body IMU signals are implicitly interpreted as body motion without explicit separation of their sources. The measured acceleration and angular velocity reflect the superposition of seat-induced vibration, passive mechanical transmission through the body, and active postural compensation, yet these contributions are not disentangled. Seat sensors, when present, are typically used to compute overall transmissibility or as additional features, rather than to construct a differential model that isolates active compensation. Furthermore, IMU-based posture and fatigue studies usually do not consider camera motion or visual sensing at all, so the sensing stack is essentially only IMU.

### 2.4. Vision-Based and RGB-D Skeletal Tracking in Vehicles

Vision-based methods have become a key pillar of driver monitoring systems, particularly for estimating head pose, gaze, and upper body posture. Early work relied on monocular RGB cameras and 2D feature tracking, but more recent approaches exploit depth sensors and learning-based pose estimators to obtain richer 3D information [59,60]. Depth and infrared cameras have been used to estimate 3D posture of vehicle occupants from interior viewpoints, achieving sub 10 cm median joint errors in constrained cabin environments and demonstrating the feasibility of in-vehicle 3D pose estimation using depth-only or depth + IR imagery [61]. In parallel, the broader RGB-D literature has shown that depth sensors and skeleton tracking enable robust people detection, gesture recognition, and multi-person pose tracking in indoor settings, where depth-based skeletons can support tasks such as action recognition and human–robot interaction [14].

Within driver monitoring, depth-based and RGB-D approaches have been proposed for head and upper body pose estimation to assess attention and readiness. For example, head pose from depth has been used to monitor driver attention under challenging illumination and occlusion conditions inside the cabin [62,63]. Other works explore 3D pose tracking of drivers and passengers using RGB or omnidirectional cameras, sometimes reconstructing 3D pose from 2D detections via triangulation or kinematic fitting [64,65]. More recently, lightweight pose-estimation frameworks such as BlazePose and MediaPipe Pose have demonstrated real-time body landmark detection from RGB images, producing 2D or 3D landmark sets that can be used to analyze posture or movement trajectories at relatively low [66,67]. These techniques have started to appear in driver and occupant monitoring prototypes, especially for tracking head orientation and facial landmarks, often combined with additional appearance-based cues for drowsiness or distraction detection.

Despite these advances, most RGB or RGB-D-based occupant monitoring studies make implicit assumptions about the stability of the camera. The camera is typically treated as rigidly fixed in a static reference frame, and any residual motion is handled, if at all, by conventional image stabilization or ignored as noise [61]. In dynamic vehicular environments, however, the camera is mounted on the vibrating structure of the cabin and therefore experiences the same low- to mid-frequency excitation as the seat and body [68]. This structural vibration results in camera jitter that induces nearly uniform apparent motion in all 3D landmarks, even when the occupant is momentarily still. Standard skeleton tracking pipelines do not distinguish between true body motion and motion caused by a vibrating camera; instead, both appear as changes in landmark coordinates.

Existing RGB-D skeleton work has extensively studied robustness to occlusion, lighting, and viewpoint, and has developed methods to fuse skeletons from multiple RGB-D cameras, perform automatic extrinsic calibration, and track multiple people in real time [10,69]. However, in most of these systems, the cameras themselves are assumed to be static in the world frame. Camera motion, when considered, is usually handled as part of visual odometry or simultaneous localization and mapping (SLAM) for scene reconstruction, not as a vibration layer that directly contaminates skeleton coordinates in a vehicle cabin. In addition, in-vehicle posture estimation methods that rely on depth cameras typically do not incorporate IMU data from the camera module, even though many modern RGB-D devices provide inertial measurements that could be used to compensate for orientation jitter.

### 2.5. IMU-RGB-D Fusion in Human Motion

The fusion of inertial and visual sensing has been extensively explored in both robotics and human motion capture. In robotics, visual–inertial odometry (VIO) and visual–inertial SLAM fuse camera images with IMU measurements to estimate the 6-DoF pose and velocity of a moving platform in real time. Filtering- and optimization-based VIO methods exploit the complementary strengths of cameras to achieve robust pose tracking in GPS-denied environments. These systems treat the IMU and camera as a rigid, calibrated unit, and focus on estimating the ego-motion of that rigid body relative to the world, not on human segment motion relative to a vibrating support [70].

In parallel, a rich literature has emerged on multimodal human motion capture that fuses body-worn IMUs with camera-based observations. Fusion schemes range from optimization-based frameworks that combine optical motion capture (OMC) with inertial motion capture (IMC) to fill gaps and improve robustness [71], to learning-based methods that integrate IMU orientation signals with 2D joint detections from RGB video or multi-view images for full-body pose reconstruction [72]. Recent surveys and systems highlight that visual–inertial fusion can significantly reduce drift and occlusion artefacts compared with pure IMU or pure vision approaches, enabling accurate motion capture with fewer cameras or fewer wearable sensors [73].

These multimodal methods typically formulate fusion as an optimization or learning problem over a kinematic skeleton. IMUs provide reliable orientation and short-term dynamics for segments, while cameras, RGB, or RGB-D supply positional constraints via 2D or 3D joint detections. Examples include systems that fuse sparse IMUs and head trackers for egocentric motion capture [74], combine wearable IMUs with multi-view cameras for improved full-body tracking [75], or integrate depth sensors and IMUs for robust performance capture with non-rigid surfaces [76]. In most cases, the environment and cameras are assumed to be static or slowly varying, and the main challenge is to resolve occlusions, reduce drift, and enforce biomechanical consistency of the reconstructed motion.

More recently, visual–inertial fusion ideas have also been applied to improve object tracking and camera-based measurement by explicitly incorporating camera kinematics into the tracking pipeline [77]. These works recognize that camera motion can confound visual tracking, and they use IMU-based pose estimates to stabilize or compensate for that motion. However, they still treat the camera–IMU rig as a single moving platform whose pose must be estimated, and they do not explicitly model a human subject seated on a separate vibrating support within the same dynamic system.

From the standpoint of the present study, existing IMU–vision and IMU-RGB-D fusion works provide strong evidence that combining inertial and visual cues improves robustness and accuracy for motion capture and pose estimation. They also show that IMU measurements from the camera itself can be used to stabilize visual information [77,78]. However, these methods typically focus on recovering the global pose of the camera or the full body in a static or slowly moving environment, and they do not address the specific structure of a seated human in a vibrating vehicle cabin. In particular, they do not treat seat vibration, body vibration, postural compensation, and camera jitter as distinct but coupled layers, nor do they define vibration-oriented metrics such as time-domain seat–body and seat–camera transmissibility, body vibration velocity, or postural compensation indices derived from differential IMU signals and motion-compensated landmark trajectories.

Thus, the IMU-RGB-D fusion framework proposed in this paper adopts a different emphasis. It leverages visual–inertial fusion not primarily to solve a general pose-estimation problem, but to decompose measured motion in a dynamic vehicular environment into platform excitation, mechanically transmitted body vibration, active postural adjustment, and camera-induced artefacts. By doing so, it connects the visual–inertial fusion literature with the WBV and in-vehicle ergonomics domains, and introduces a set of metrics specifically designed to isolate seated human body vibration and postural compensation under realistic dynamic excitation.

## 3. Materials and Methods

### 3.1. Visual–Inertial Fusion Framework for Isolating Seated Body Vibration

**Figure 1:**
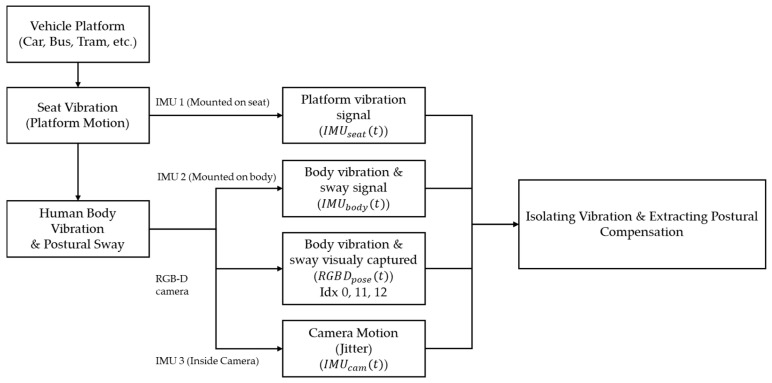
Visual–inertial fusion framework diagram.

This section outlines the IMU-RGB-D fusion framework proposed to isolate seated human body vibration in dynamic environments. The used IMU sensor is WiTMotion WT901BLECL (Shenzhen Co., Ltd., Shenzhen, China) that also used to obtain real-time feedback in a former study [79,80]. Meanwhile, the Intel^®^ RealSense™ Depth Camera D457 (Intel Corporation, Santa Clara, CA, USA) is used as the RGB-D camera sensor [81]. Rather than treating all sensor outputs as a single motion signal, the framework explicitly decomposes motion into seat vibration and body vibration and postural sway, through the observed vehicle’s platform, and body motion. By separating these components and then recombining them through defined metrics, the framework yields an estimate of isolated seated body vibration together with indices of how strongly the body compensates through posture. The proposed framework diagram is visualized in Figure 1.

Vibration from the vehicle, acting as the dynamic environment in this study, is first captured by the seat IMU, which provides the platform vibration signal $IMU_{seat}(t)$ containing linear acceleration and angular velocity. The body IMU then measures the resulting body vibration and sway $IMU_{body}(t)$. In parallel, the camera IMU records camera motion, while the RGB-D camera provides pose estimates $RGBD_{pose}(t)$ for selected landmarks. Three core landmarks are used in this work, which are the nose, left shoulder, and right shoulder, to analyze postural sway under dynamic vibration.

On the IMU side, seat acceleration and angular velocity are used to construct a passive body prediction, representing the motion that would occur if the torso simply followed the seat with a fixed gain. Subtracting this prediction from the measured body signal yields a rotational postural compensation signal, from which the rotational Postural Compensation Index (PCI) is obtained as a sliding RMS. The same IMU signals also support computation of seat–body transmissibility by obtaining VTR and body vibration velocity v(t) via band-pass filtering, fast Fourier transform (FFT)-based spectral integration, and inverse FFT.

On the visual side, camera motion is first compensated using the camera IMU before 3D landmarks are transformed into the world coordinate frame. A static reference pose is then estimated over a short calibration interval, and translational postural compensation is obtained by subtracting this anchored reference from the world-frame landmark trajectories. The translational PCI is defined as the sliding RMS of this compensation signal for each landmark.

### 3.2. Sensing Configuration and Coordinate Systems

This study uses a multimodal sensing setup that combines three inertial measurement units (IMUs) with an RGB-D camera to capture seat vibration, body vibration, postural sway, and camera jitter, as well as visually observed body motion, in a unified framework. This proposed framework considers where the sensors are placed, what each one measures, and how all measurements are expressed in consistent coordinate frames for subsequent fusion.

#### 3.2.1. Multimodal Sensing Setup

Three IMUs are deployed in this study, which are the seat IMU, body IMU, and camera IMU. Seat IMU provide tri axial accelerometer and gyroscope that are rigidly attached to the seat frame. Its signal, denoted as $IMU_{seat}(t)$, represents platform vibration transmitted from the vehicle structure to the seat. The second IMU is the body IMU. It is mounted on the chest, specifically the anterior thorax, near the mid sternum. The body IMU signal $IMU_{body}(t)$ captures body vibration and postural sway relative to this neutral seated posture. The third is a camera IMU. It is integrated in the RGB-D Intel RealSense D457. It provides linear acceleration and angular velocity, which can be calculated as orientation quaternion $IMU_{cam}(t)$, which characterize jitter camera motion and later support inverse orientation of RGB-D landmarks.

The RGB-D camera is mounted on the tram interior with a suction mount at the window and faces the participant. Colour and depth streams are acquired at the video rate. Each colour frame is processed on a host computer using the MediaPipe pose library (Google LLC), and the aligned depth map is then used to reconstruct 3D positions of three core landmarks, which are the nose, left shoulder, and right shoulder, forming $RGBD_{pose}(t)$ using the camera intrinsic parameter. These landmarks are the basis for translational PCI and anthropometric analysis. All three IMUs run at ≈100 Hz, while the RGB-D camera operates at 15–30 fps. Each IMU packet and RGB-D frame is timestamped using the same system clock, enabling alignment on a common time axis.

#### 3.2.2. Coordinate Frames

In this study, IMU signals are interpreted primarily in their own local sensor frames, rather than being fully mapped into a global world frame. The initialization is applied prior to the whole data being recorded. All IMU axes are approximately aligned with each other in neutral posture, so the gravity direction can be consistent across sessions. It kept the comparisons of magnitudes, and axis-wise contributions are meaningful. For the RGB-D side, an explicit world frame $F_{w}$ is defined through extrinsic calibration of the camera using a checkerboard with 7 × 5 inner corners and 0.03 m square size. The world axes are chosen as x for lateral left-right, y for vertical up-down, and z for anteroposterior forward-backward relative to the seat.

Landmarks are first reconstructed in the camera coordinate, then reverse orientation is applied using the camera IMU quaternion, and then the checkerboard extrinsics are used to map them into $F_{w}$. The world coordinate landmark trajectories $p_{l}^{w}(t)$ for the nose, left shoulder, and right shoulder are subsequently used for translational PCI, axis-wise analysis, and anthropometric consistency, while IMU based metrics VTR, vibration velocity, and rotational PCI are derived from the IMU frames.

#### 3.2.3. Data Matrix Representation and Calibration Metadata

All described signals are stored in a single synchronized time log file with one row per time sample and multiple columns corresponding to IMUs, RGB-D landmarks, and derived distances. Let the $t_{i}$ denote the timestamp of the T(t)-th sample, t=1,…,N. For each sample, we defined a measurement vector $P\left(t\right)\in R^{D}$ that concatenates all recorded channels in the groups. For the seat and body IMU, we obtained linear acceleration a and angular velocity ω that contain 3 coordinates. Analogous to the seat and body IMU, from the camera IMU, we obtained a and ω, plus a quaternion q that contains 4 coordinates calculated from its a and ω using a Madgwick filter [82,83]. Then, the 3D position of nose, left shoulder (lsh) and right shoulder (rsh) that contains 3 coordinates are denoted as $p_{nose}\left(t\right),\;p_{lsh}\left(i\right),\;p_{rsh}\left(i\right)$. Then, two scalar anthropometric distance measurements are logged per sample as $d_{sh_{meas}}(t)$ for shoulder width and $d_{nm_{meas}}(t)$ for nose–mid-shoulder distance. The distance is calculated using 3D Euclidean distance between 2 points, reconstructed from RGB-D landmarks. The nominal shoulder width and nose–mid-shoulder distances were measured physically with a tape measure and stored constant across all time samples in a given session as $d_{sh_{real}}$ for shoulder width and $d_{nm_{real}}$ for nose–mid-shoulder distance. Collecting all components, each row vector is written as Equation (1).

(1) $$\begin{array}{ll} p_{i}= & [T(t),a_{{body}_{x}}(t),a_{{body}_{y}}(t),a_{{body}_{z}}(t),\omega_{{body}_{x}}(t),\omega_{{body}_{y}}(t),\omega_{{body}_{z}}(t),\\ & a_{{seat}_{x}}(t),a_{{seat}_{y}}(t),a_{{seat}_{z}}(t),\omega_{{seat}_{x}}(t),\omega_{{seat}_{y}}(t),\omega_{{seat}_{z}}(t),\\ & a_{{cam}_{x}}(t),a_{{cam}_{y}}(t),a_{{cam}_{z}}(t),\omega_{{cam}_{x}}(t),\omega_{{cam}_{y}}(t),\omega_{{cam}_{z}}(t),\\ & q_{1}(t),q_{2}(t),q_{3}(t),q_{4}(t),\\ & p_{{nose}_{x}}(t),p_{{nose}_{y}}(t),p_{{nose}_{z}}(t),\\ & p_{{lsh}_{x}}(t),p_{{lsh}_{y}}(t),p_{{lsh}_{z}}(t),\\ & p_{{rsh}_{x}}(t),p_{{rsh}_{y}}(t),p_{{rsh}_{z}}(t)\\ & d_{sh_{meas}}(t),d_{nm_{meas}}(t),d_{sh_{real}},d_{nm_{real}}] \end{array}$$

and the full dataset is represented as a $P={\left[\begin{array}{c} p_{1},p_{2},\ldots ,p_{N} \end{array}\right]}^{T}$.

In addition to the time series matrix P, camera calibration metadata are stored and used for analysis. The error measurement is provided to ensure the accuracy of 3D landmark reconstruction. It is done by subtracting the measured 2 valid points distance from the checkerboard and the estimated distance from reconstructed RGB-D points of the checkerboard.

### 3.3. Signal Preprocessing and Camera Reverse Orientation

Building on the data matrix P, this study involves multimodal signals cleaning, normalizing, and stabilizing before vibration and postural metrics are computed. The objectives are to reject clearly invalid samples, ensure that all modalities share the same valid time instant, isolate dynamic vibration components, and remove camera orientation jitter from the RGB-D landmarks.

#### 3.3.1. Global Frame Level Quality Mask

After temporal alignment, the i-th row $p_{i}$ contains all measurements at time $T_{i}$. For each index i, a binary global mask $m_{i}\left(t\right)\in \{0,1\}$ is defined as Equation (2).

(2) $$m_{i}\left(t\right)=m_{IMU_{i}}\left(t\right)\wedge m_{RGBD_{i}}(t)$$

where $m_{IMU_{i}}(t)$ encodes the validity of seat and body IMUs, and $m_{RGBD_{i}}(t)$ encodes the validity of 3D landmarks. Only samples with $m_{i}\left(t\right)=1$ are retained for subsequent analyses. All vibration, PCI, and anthropometric metrics are computed on this curated subset. The resulting exclusion rates and rejection causes are reported in Section 4.2, specifically in Table 1. In our experiments, most rejected frames were due to RGB-D depth loss or depth spikes, while IMU-related rejections were rare.

#### 3.3.2. IMU Validation and Dynamic Component

From $p_{i}$, the body and seat accelerations are denoted as $a_{body}\left(t\right)$, consisting of $a_{{body}_{x}}\left(t\right),a_{{body}_{y}}\left(t\right),$ and $a_{{body}_{z}}\left(t\right)$, and $a_{seat}(t)$, consisting of ${\;a}_{{seat}_{x}}\left(t\right),a_{{seat}_{y}}\left(t\right),$ and $a_{{seat}_{z}}\left(t\right)$. Meanwhile, the body and seat angular velocities are denoted as $\omega_{body}\left(t\right)$, consisting of $\omega_{{body}_{x}}\left(t\right),\omega_{{body}_{y}}\left(t\right),\omega_{{body}_{z}}\left(t\right)$, and $\omega_{seat}(t)$, consisting of $\omega_{{seat}_{x}}\left(t\right),\omega_{{seat}_{y}}\left(t\right),\omega_{{seat}_{z}}\left(t\right)$. For IMU validation, only acceleration magnitudes are thresholded, since gross sensor faults or impacts manifest first in acceleration. The norms $a_{body}\left(t\right)=\left\|a_{body}\left(t\right)\right\|$ and $a_{seat}\left(t\right)=\|a_{seat}\left(t\right)\|$ are expected to remain close to 1g with moderate fluctuations in a normal tram ride. A conservative upper bound $a_{th}$ = 30 m/s^2^ is applied to both norms to flag unrealistic spikes. This threshold is set well above the accelerations expected from WBV in rail vehicles and above any values observed in preliminary recordings, while remaining far below the IMU full-scale range. It therefore removes only implausible outliers caused by sensor glitches or decoding errors, without clipping genuine vibration events. The IMU validity flag is defined as Equation (3).

(3) $${m_{IMU}}_{i}\left(t\right)=1\left(a_{body}\left(t\right)<a_{th}\right)\wedge 1\left(a_{seat}\left(t\right)<a_{th}\right)$$

where 1(⋅) is the indicator function. Samples where either norm exceeds $a_{th}$ are rejected, with angular velocities $\omega_{body}\left(t\right)$ and $\omega_{seat}(t)$ are implicitly validated through this same mask.

To focus on dynamic vibration, static components, which are gravity and slow drift, are removed in a metric-specific manner. For RMS-based time domain metrics, a local is subtracted in each sliding window so that only fluctuations around the local baseline contribute to the RMS. For body vibration velocity v(t), a differential acceleration denoted as $a_{diff}\left(t\right)=a_{body}\left(t\right)-k\cdot a_{seat}(i)$ is performed with a fixed gain k representing an approximate passive seat–body transfer, then passed through a 1–20 Hz bandpass filter before FFT-based spectral integration. This band emphasizes the frequency range relevant to WBV while attenuating very low frequency drift and high frequency noise. Unless stated otherwise, the 1–20 Hz band is used here as a practical WBV-dominant working range for seated in-vehicle vibration analysis, while suppressing quasi-static components (<≈1 Hz) and high-frequency sensor or mount noise. This choice follows the common practice of frequency-selective evaluation in WBV standards ISO 2631/AS 2670.1 [19], where vibration is assessed with axis-wise treatment in the seat-relative world frame (x lateral, y vertical, z anteroposterior) rather than as a single unfiltered signal. Importantly, the proposed fusion and decomposition framework is not restricted to this band. The cut-off frequencies can be adjusted for other vehicles and operating conditions with different vibration spectra, and the decomposed signals can serve as inputs for standard frequency-weighted indices. Validated and bandpass filtered IMU signals thus provide the dynamic excitation from the seat and response from the body used to compute VTR, vibration velocity. Meanwhile, the rotational PCI is computed by implementing 2 s sliding RMS on $\omega_{diff}\left(t\right)=\omega_{body}\left(t\right)-k\cdot \omega_{seat}(i)$.

#### 3.3.3. RGB-D Landmark Validation

The 3D landmark positions at time T(t)—denoted as $p_{nose}(t)$, consisting of ${p_{nose}}_{x}\left(t\right),\;{p_{nose}}_{y}\left(t\right),{p_{nose}}_{z}(t)$; $p_{lsh}(t)$, consisting of ${p_{lsh}}_{x}\left(t\right),\;{p_{lsh}}_{y}\left(t\right),{p_{lsh}}_{z}(t)$; and $p_{rsh}(t)$ consisting of ${p_{rsh}}_{x}\left(t\right),\;{p_{rsh}}_{y}\left(t\right),{p_{rsh}}_{z}(t)$—are then summarized as $p_{l}$. At acquisition time, these points are reconstructed in the camera frame from 2D pixels and depth values. In addition to depth validity, landmark reliability is screened using MediaPipe Pose confidence scores. Frames are accepted only when the detected nose and shoulder landmarks satisfy minimum visibility thresholds as provided by MediaPipe, to reject occlusions and unstable detections before depth gating. In a dynamic tram environment, depth is sometimes missing and throws back a NaN/zero or out of a plausible range due to occlusion, reflections, or loss of stereo correspondence near windows. To guard against such artefacts, the RGB-D validity flag is defined as Equation (4).

(4) $$m_{RGBD}\left(t\right)=\prod_{l\in \left\{nose,\;lsh,\;rsh\right\}}1\left(p_{l}\left(i\right)\;is\;true\right)\wedge 1\left(z_{l}\left(i\right)\in \left[z_{min},z_{max}\right]\right)$$

where $z_{l}(i)$ is the depth component of landmark l in the camera frame, and the depth range $\left[z_{min},z_{max}\right]=[0,2]$ m is chosen as physically plausible for the seated human body. Any sample where one or more landmarks are underlined or outside this depth band is rejected for all downstream metrics. Applying both $m_{IMU}(i)$ and $m_{RGBD}(i)$ yields a curated subset of rows $\left\{p_{i}:m\left(t\right)=1\right\}$ in which IMU signals and landmarks are simultaneously reliable.

#### 3.3.4. Camera Reverse Orientation and World Coordinate Landmarks

Even when depth values are valid, the RGB-D landmarks are still affected by camera motion since small rotations of the camera cause apparent motion of all 3D points, even if the body remains still. To separate true body motion from camera jitter, the orientation quaternion is denoted as $q\left(t\right)\in p_{i}$ consisting of $q_{1}\left(t\right),\;q_{2}\left(t\right),\;q_{3}\left(t\right),q_{4}\left(t\right)$ is used to reverse the compensated landmarks. The quaternion $q\left(t\right)$ is first converted to a rotation matrix $R_{cam}\left(t\right)\in SO(3)$ representing the camera orientation relative to an internal inertial frame. For each landmark $p_{l}(t)$ expressed in camera coordinate, a motion-compensated landmark is obtained as Equation (5).

(5) $$p_{l}^{dejit}\left(t\right)=R_{cam}{\left(t\right)}^{T}p_{l}(t)$$

which effectively inverting orientation of the camera motion, so that $p_{l}^{dejit}\left(t\right)$ is expressed in an inertial camera coordinate. Then, by using the extrinsic parameter $R_{ext},t_{ext}$ From the calibration step, the reversed landmarks are then mapped into the tram world coordinate as Equation (6).

(6) $$p_{l}^{w}\left(t\right)=R_{ext}\;p_{l}^{dejit}\left(t\right)+t_{ext},\;l\in \{nose,lsh,rsh\}$$

In the remainder of this paper, $p_{l}^{w}(t)$ are used as the world frame landmark trajectories, and the notation $p_{l}(t)$ is abused to refer to these world coordinates unless explicitly stated otherwise.

### 3.4. Vibration Decomposition and Core Metrics

This section formalizes how vibration is decomposed into three layers, which are seat, body, and camera, and how the core vibration metrics are defined; those are seat–body VTR, seat–camera VTR, and body vibration velocity. The proposed separation is a physically grounded kinematic decomposition rather than a detailed biomechanical model. In the WBV-dominant frequency band considered in this study, the instrumented seat base and the camera mount are treated as rigid bodies with repeatable mounting orientation, while the body-mounted IMU is used as a proxy for the upper-torso segment under a quasi-rigid approximation. The subject is seated with continuous body–seat contact, so the measured torso motion reflects the combined effect of seat excitation, passive transmission through the contact interface, and postural adjustments about a moving base. These assumptions enter the framework through the common world-frame alignment and consistent sensor mounting; therefore, the derived differential signals and VTR/PCI metrics are interpreted as relative motion indicators under seated constraints rather than direct physiological activation measures.

All quantities are derived from the pre-processed and time-aligned signals contained in the data matrix P. Let the discrete time index i=1,…,N denote the sample at time T(t). From $p_{i}$, the raw accelerations are $a_{body}\left(t\right)$, $a_{seat}\left(t\right)$, and the camera IMU acceleration is denoted as $a_{cam}\left(t\right)$, consisting of ${\;a}_{{cam}_{x}}\left(t\right),a_{{cam}_{y}}\left(t\right),a_{{cam}_{z}}\left(t\right)$, where $a_{cam}\left(t\right)$ is obtained from the camera IMU channel associated with the quaternion q(t).

#### 3.4.1. Dynamic Excitation and Response Layers

Within this framework, the three vibration layers are defined as seat excitation, body response, and camera vibration. Seat excitation $a_{seat}\left(t\right)$ represent the dynamic excitation transmitted from the rail wheel interface through the tram structure to the seat. Body response $a_{body}\left(t\right)$ represents the dynamic response of the upper torso to seat excitation, as measured by the body IMU. Camera vibration $a_{cam}\left(t\right)$ represents the dynamic vibration experienced by the camera module and its mounting structure. This decomposition allows the proposed framework to isolate seated body vibration relative to both the seat and the camera, rather than conflating all motion into a single measure. It assumes consistent IMU mounting across sessions with fixed orientation relative to the seat/body/camera rigid bodies, and axis alignment via the common world-frame transformation in preprocessing. In practice, we used rigid mounts and repeatable placements and treated k as an approximate passive seat–body transfer factor. The gain k is introduced to remove the component of body acceleration attributable to seat excitation. In practice, k is selected from a pre-experimental passive-following condition (or low-activity segments) by minimizing the residual energy between response $a_{body}$ and a scaled $a_{seat}$, and it is treated as a pragmatic scaling parameter rather than a subject-specific biomechanical constant.

#### 3.4.2. Seat–Body Vibration Transmission Ratio (VTR)

To quantify how strongly seat vibration is transmitted to the body in the time domain, a sliding RMS is computed over a window W(i) centred at sample i, with a duration ΔT ≈ 2 s. A 2 s window provides a balance between temporal resolution and statistical stability. At the sampling rate used in this study, it contains sufficient samples and spans multiple cycles across the dominant WBV band (1–20 Hz), reducing sensitivity to instantaneous spikes while still tracking changes during acceleration and braking segments. Shorter windows increase temporal responsiveness but yield noisier RMS-based metrics, whereas longer windows improve smoothness at the cost of smearing transient events. For consistency, the same window length is used for all RMS-based metrics reported in this work. The seat and body RMS magnitude are defined in Equation (7).

(7) $$RMS_{seat}\left(i\right)=\sqrt{\frac{1}{\left|W\left(i\right)\right|}\Sigma_{j\in W\left(i\right)}{\left\|a_{seat}\left(t\right)\right\|}^{2}},\;RMS_{body}\left(i\right)=\sqrt{\frac{1}{\left|W\left(i\right)\right|}\Sigma_{j\in W\left(i\right)}{\left\|a_{body}\left(t\right)\right\|}^{2}}\;$$

Thus, the seat–body VTR is then given by Equation (8).

(8) $$VTR\left(i\right)=\frac{RMS_{body}\left(i\right)}{RMS_{seat}\left(i\right)}$$

Value $VTR\left(i\right)\approx 1$ indicates that the body experiences vibration levels comparable to the seat with near passive transmission, $VTR\left(i\right)<1$ indicates attenuation by the body with damping possibility via soft tissues and posture, and $VTR\left(i\right)>1$ indicates amplification, typically associated with local resonance or active motion.

#### 3.4.3. Seat–Camera VTR for Visual Stability

An analogous ratio is defined between the camera and the seat to characterize visual sensor stability. Denoted as $VTR_{cam}(i)$, its ratio comparing $RMS_{cam}\left(i\right)$ is obtained from $\sqrt{\frac{1}{\left|W\left(i\right)\right|}\Sigma_{j\in W\left(i\right)}{\left\|a_{cam_{rel}}\left(t\right)\right\|}^{2}}$ with $RMS_{seat}\left(i\right)$, where $a_{cam_{rel}}\left(t\right)$ is the relative camera acceleration to the seat, by calculating $a_{cam}\left(t\right)-a_{seat}\left(t\right)$. Here, $VTR\left(i\right)\approx 1$ implies that the camera experience vibration comparable to the seat, while $VTR_{cam}\left(i\right)>1$ indicates that the camera mount amplifies certain vibration components because of cantilever behaviour on the window, increasing the risk of skeleton distortion if camera jitter is not compensated. The metric is later related to the stability of RGB-D landmarks and the need for IMU based reverse orientation.

#### 3.4.4. Body Vibration Velocity

While acceleration is directly measured, vibration velocity is often more closely related to subjective comfort and biodynamic standards. To estimate body vibration velocity while discounting purely passive following of the seat, a differential acceleration signal is formed as $a_{diff}\left(t\right)$. To focus on dynamic components, a bandpass operator B{⋅} is applied to the differential acceleration signal. For notational simplicity, the resulting dynamic accelerations are denoted as ${\tilde{a}}_{diff}\left(t\right)=B\left\{a_{diff}\left(t\right)\right\}$. In practice, B{⋅} combines offset removal and bandpass filtering in the 1–20 Hz range, which covers the dominant WBV band for seated humans while suppressing drift and high-frequency noise.

The discrete-time FFT of ${\tilde{a}}_{diff}\left(t\right)$ is denoted by $A_{diff}(f)=F\{{\tilde{a}}_{diff}(i)\}$, with frequency variable f. To obtain the corresponding velocity spectrum, frequency domain integration is applied for frequencies above a minimum cutoff $f_{min}$ to avoid numerical issues at very low frequencies, denoted as Equation (9).

(9) $$V\left(f\right)=\frac{A_{diff}\left(f\right)}{j2\pi f},\;f\geq f_{min}$$

and V(f) is set to zero for $f<f_{min}$. In this work, vibration velocity v(t) is obtained by first band-pass filtering the differential acceleration signal in the 1–20 Hz range and then performing spectral integration only above a low-frequency cutoff $f_{min}$ = 0.8 Hz. Very low frequencies are dominated by quasi-static trends and slow postural drifts rather than perceived vibration, and are known to cause numerical drift when divided by f in the frequency domain. In line with AS 2670-1, which considers the 0.5–80 Hz band as relevant for WBV comfort and health, and with ride-comfort studies reporting that the main comfort-relevant vibration band for seated occupants with a seat back lies approximately between 0.8 and 10 Hz [84,85], the cutoff 0.8 Hz is chosen as a physiology-informed compromise. It attenuates quasi-static components while preserving the dominant ride-comfort frequencies around the first pelvis–spine resonance. Thus, the time domain body vibration velocity is then obtained via inverse transform as Equation (10).

(10) $$\left(t\right)=F^{-1}\{V\left(f\right)\}$$

In summary, $a_{seat}\left(t\right),a_{body}\left(t\right),$ and $a_{cam}\left(t\right)$ define the three principal vibration layers for seat excitation, body response, and camera vibration. From these, the time domain seat–body VTR, seat–camera VTR, and velocity-based body vibration metric provide complementary views of how vehicle vibration is transmitted, amplified, or attenuated by both the human body and the visual sensing system.

### 3.5. Rotational and Translational Postural Compensation Index (PCI)

The Postural Compensation Index (PCI) quantifies relative postural adjustment in response to seat vibration, beyond purely passive motion. In this study, PCI is used as a physically grounded proxy of relative postural adjustment under seat excitation. However, it does not uniquely separate active neuromuscular control from passive body–seat mechanical dynamics, which may contribute jointly to the observed relative motion. PCI is introduced as a study-specific metric to quantify active postural compensation and is not defined in existing ISO vibration standards. Two complementary forms are used in this framework, which are rotational PCIs derived from seat and body angular velocities, and translational PCI derived from world frame trajectories of the head and shoulders. Both indices are computed as sliding RMS values over a temporal window W(i) of approximately 2 s centred on sample i, consistent with the RMS windows used for VTR and vibration velocity.

#### 3.5.1. Rotational PCI ($PCI_{rot}$)

From the data vector $p_{i}$, the body and seat angular velocities are denoted as $\omega_{body}(t)$ consisting of $\omega_{body_{x}}\left(t\right),\;\omega_{body_{y}}\left(t\right),\;{\;\omega }_{body_{z}}(t)$ and $\omega_{seat}(t)$ consisting of $\omega_{{seat}_{x}}\left(t\right),{\;\omega }_{{seat}_{y}}\left(t\right),{\;\omega }_{{seat}_{z}}(t)$, respectively. The rotational postural compensation signal is defined as the difference between body and seat angular velocities, denoted by $\omega_{diff}\left(t\right)$. Intuitively, $\omega_{diff}\left(t\right)$ represents how strongly the torso rotates relative to the passive body following the seat, such as the component of motion that cannot be explained by the seat moving as a rigid base. We noted that $\omega_{diff}\left(t\right)$ may reflect both passive compliance/rocking of the body–seat interface and active corrective rotations. Therefore, $PCI_{rot}$ is interpreted as a relative rotation index rather than a direct measure of neuromuscular activation. The rotational PCI at sample i is then defined as the RMS magnitude of $\omega_{diff}\left(t\right)$ over the window W(i), as can be seen in Equation (11).

(11) $$PCI_{rot}\left(i\right)=\sqrt{\frac{1}{\left|W\left(i\right)\right|}\Sigma_{j\in W\left(i\right)}{\left\|\omega_{diff}\left(t\right)\right\|}^{2}}$$

Low values of $PCI_{rot}(i)$ indicate that the body rotates almost rigidly with the seat by nearly passive behaviour, whereas pronounced peaks indicate active reorientation of the torso, such as leaning, bracing, or corrective angular responses, on top of the base vibration.

#### 3.5.2. Translational PCI ($PCI_{trans}$)

For translational motion, PCI is computed from world frame trajectories of the three key landmarks, which are the nose, left shoulder, and right shoulder. After reverse orientation as mentioned in Section 3.4, each landmark l∈{nose, lsh, rsh} has a world frame position $p_{l}^{w}(t)$ containing $p_{l_{x}}^{w}\left(t\right),\;{\;p}_{y}^{w}\left(t\right),\;{\;p}_{l_{z}}^{w}(t)$ at time index t. A neutral reference posture for each landmark is then obtained by averaging over a short static interval $T_{ref}$, as can be seen in Equation (12).

(12) $${\bar{p}}_{l}^{w}=\frac{1}{\left|T_{ref}\right|}\Sigma_{i\in T_{ref}}p_{l}^{w}(t)$$

The translational compensation of landmark l is then defined as the deviation from its reference, denoted as $c_{l}\left(t\right)=p_{l}^{w}\left(t\right)-{\bar{p}}_{l}^{w}$. Thus, an RTV can be obtained by finite differences between consecutive samples as $v_{l}\left(i\right)=\frac{c_{l}\left(t\right)-c_{l}\left(t-1\right)}{\Delta T}$, where $\Delta T=T_{i}-T_{i-1}$ is the sampling interval. This RTV captures how fast each landmark translates relative to its neutral position. The translational for landmark l is then defined, analogously to the rotational case, as the sliding RMS of the RTV magnitude over window W(i), denoted in Equation (13).

(13) $$PCI_{trans}\left(i\right)=\sqrt{\frac{1}{\left|W\left(i\right)\right|}\Sigma_{j\in W\left(i\right)}{\left\|v_{l}\left(j\right)\right\|}^{2}},\;l\in \{nose,lsh,rsh\}$$

In this formulation, $PCI_{nose}(i)$ quantifies translational compensation of the head, while $PCI_{lsh}\left(i\right)$ and $PCI_{rsh}(i)$ quantify compensation of the left and right shoulder. Comparing these three indices reveals segment-specific strategies. In several cases, the head may exhibit higher lateral compensation than the shoulders, or the shoulders may show stronger anteroposterior sway in response to acceleration and braking. Such segmental information is not accessible from a single chest IMU alone, and is a key advantage of fusing IMU with RGB-D landmarks in the proposed framework.

### 3.6. Axis-Wise Anisotropy and Anthropometric Consistency

In addition to global magnitudes VTR and PCI, the proposed framework analyzes the directional distribution of vibration and postural compensation, as well as the anthropometric consistency of RGB-D landmarks. The former reveals anisotropy in how the body moves, while the latter checks whether the fused IMU-RGB-D pipeline preserves basic body geometry under vibration. Let $I=\{t:m\left(t\right)=1\}$ denote the set of valid samples after quality masking.

#### 3.6.1. Axis-Wise Energy Distribution of Differential Body Vibration and PCI

For the direction analysis of active body response, the proposed framework uses the bandpass filtered differential acceleration between body and seat rather than body acceleration alone. As mentioned, the dynamic differential acceleration is denoted as ${\tilde{a}}_{diff}\left(t\right)$, containing ${\tilde{a}}_{diff_{x}}\left(t\right),\;{\tilde{a}}_{diff_{y}}\left(t\right),\;{\tilde{a}}_{diff_{z}}\left(t\right)$. To quantify anisotropy in this relative response, the axis-wise energy of ${\tilde{a}}_{diff}\left(t\right)$ is computed over all valid subsets I. Then the mean offset is removed from each axis using ${\tilde{a}}_{diff}\left(t\right)-\frac{1}{\left|I\right|}\Sigma_{i\in I}\;{\tilde{a}}_{diff}\left(t\right)$, denoted as ${\tilde{a}}_{diff}^{dyn}\left(t\right)$, containing ${\tilde{a}}_{{diff}_{x}}^{dyn}\left(t\right),\;{\tilde{a}}_{{diff}_{y}}^{dyn}\left(t\right),\;{\tilde{a}}_{{diff}_{z}}^{dyn}\left(t\right)$. It is done to ensure that subsequent RMS and axis-wise energy measures reflect true dynamic vibration rather than static contribution from gravity, sensor bias, or quasi-static posture. A sliding RMS is then computed for each axis over a window W(i) of duration ΔT≈2 s using ${RMS}_{x}\left(i\right)=\sqrt{\frac{1}{\left|W\left(i\right)\right|}\Sigma_{j\in W\left(i\right)}{\left\|{\tilde{a}}_{{diff}_{x}}^{dyn}\left(t\right)\right\|}^{2}}$, and similarly ${RMS}_{y}\left(i\right)$ and ${RMS}_{z}\left(i\right)$. A scalar summary for each axis is obtained by averaging these RMS values over all valid subset as can be seen in Equation (14).

(14) $$R_{x}=\frac{1}{\left|I\right|}\Sigma_{i\in I}RMS_{x}\left(i\right),\;{\;R}_{y}=\frac{1}{\left|I\right|}\Sigma_{i\in I}RMS_{z}\left(i\right),\;{\;R}_{z}=\frac{1}{\left|I\right|}\Sigma_{i\in I}RMS_{z}(i)$$

The axis-wise contribution of the differential body vibration is then defined in Equation (15).

(15) $$P_{x}^{body}=\frac{R_{x}}{R_{x}+R_{y}+R_{z}}\times 100\%,\;\;P_{y}^{body}=\frac{R_{y}}{R_{x}+R_{y}+R_{z}}\times 100\%,\;\;P_{z}^{body}=\frac{R_{z}}{R_{x}+R_{y}+R_{z}}\times 100\%$$

Analogous axis-wise energy ratios are computed for the translational PCI velocities of each landmark $v_{l}\left(i\right)$, denoted as Equation (16).

(16) $$\begin{array}{l} E_{x}^{l}=\frac{1}{\left|W\left(i\right)\right|}\Sigma_{j\in W\left(i\right)}{\left\|v_{l_{x}}\left(i\right)\right\|}^{2},\;\\ E_{y}^{l}=\frac{1}{\left|W\left(i\right)\right|}\Sigma_{j\in W\left(i\right)}{\left\|v_{l_{y}}\left(i\right)\right\|}^{2},\;E_{z}^{l}=\frac{1}{\left|W\left(i\right)\right|}\Sigma_{j\in W\left(i\right)}{\left\|v_{l_{z}}\left(i\right)\right\|}^{2} \end{array}$$

with corresponding percentages as Equation (17).

(17) $$P_{x}^{l}=\frac{E_{x}^{l}}{E_{x}^{l}+E_{y}^{l}+E_{z}^{l}}\times 100\%,\;\;P_{y}^{l}=\frac{E_{y}^{l}}{E_{x}^{l}+E_{y}^{l}+E_{z}^{l}}\times 100\%,\;\;P_{z}^{l}=\frac{E_{z}^{l}}{E_{x}^{l}+E_{y}^{l}+E_{z}^{l}}\times 100\%$$

Comparing $P_{x,y,z}^{body}$ with $P_{x,y,z}^{l}$ across nose and shoulders reveals directional strategies of postural control.

#### 3.6.2. Anthropometric Distance Definitions

To assess whether RGB-D landmarks remain geometrically consistent under vibration, two anthropometric distances are defined in the world frame trajectories, which are shoulder-width and nose–mid-shoulder distance. Shoulder width is calculated using the 3D Euclidean distance between $p_{lsh}^{w}\left(t\right)$ and $p_{rsh}^{w}\left(t\right)$ and stated as $d_{sh_{meas}}(t)$. Analogous, the nose–mid-shoulder $d_{{nm}_{meas}}(t)$ is also calculated using the 3D Euclidean distance between $p_{nose}^{w}\left(t\right)$ and $p_{mid}^{w}\left(t\right)$, where $p_{mid}^{w}\left(t\right)=\frac{p_{lsh}^{w}\left(t\right)+p_{rsh}^{w}\left(t\right)}{2}$. The corresponding groundtruth values $d_{sh_{real}}$ and $d_{nm_{real}}$ are measured with a tape before the experiment and stored as constant scalars in each row $p_{i}$. Shoulder width is expected to be nearly constant anthropometrically, whereas nose–mid-shoulder distance can vary moderately with head posture. These distances provide complementary constraints for seated upper body analysis. Shoulder width is a time-invariant anthropometric length useful for detecting tracking or calibration artefacts, while the nose–mid-shoulder distance links head-to-torso configuration and can vary moderately with genuine head/neck posture.

#### 3.6.3. Anthropometric Deviations and Interpretation

Anthropometric deviations are defined as the difference between the measured and ground truth distances as denoted in Equation (18).

(18) $$e_{sh}\left(t\right)=d_{sh_{meas}}\left(t\right)-d_{sh_{real}},\;e_{nm}\left(t\right)={d_{nm}}_{meas}\left(t\right)-d_{{nm}_{real}}$$

For an ideal position, $e_{sh}\left(t\right)$ would remain close to zero, with variations mainly due to pose estimation noise and very small shoulder movements. In practice, |$e_{sh}\left(t\right)$| reflects a combination of camera-to-world calibration accuracy, residual camera jitter still not removed by the motion compensation system, and MediaPipe landmark noise at the shoulders. Because shoulder width is an almost constant anthropometric quantity, $e_{sh}\left(t\right)$ serves as a stability check for the fused pipeline.

In contrast, $e_{nm}\left(t\right)$ is influenced not only by sensing and calibration but also by genuine head motion. Deviations in ${d_{nm}}_{meas}\left(t\right)$ arise when the participant flexes or extends the neck, or slightly nods in response to vibration or visual cues. Consequently, $e_{nm}\left(i\right)$ is interpreted as a mixed indicator of head-neck dynamics and sensitivity of the nose landmark to residual jitter and detection noise.

Together, the axis-wise energy ratios and anthropometric deviation metrics complement VTR, vibration velocity, and PCI. Axis-wise analysis reveals direction control strategies, while anthropometric consistency tests whether the IMU-RGB-D fusion produces geometrically stable skeletons in a vibrating vehicle, which is crucial for reliable posture-based monitoring in dynamic environments.

## 4. Experimental Results

The proposed framework is instantiated in a representative case study, which is an in-service urban tram operating under normal conditions with four-fold data acquisitions. All results are presented so that the steps of the framework are clear. It involves the acquisition of multimodal signals, quality control and preprocessing, vibration decomposition into seat, camera, and body motion, and extraction of metrics that characterize isolated seated body vibration and postural compensation.

### 4.1. Experimental Environment and Fusion Sensor Configuration

Experiments were conducted inside an in-service urban tram running on its regular route, so that the recorded signals reflect a realistic dynamic vehicular environment. A healthy adult (a member of the research team) sat on an instrumented passenger seat and was instructed to maintain a natural seated posture, so that measured motion was dominated by vehicle-induced vibration and spontaneous postural responses rather than deliberate movements. This dataset was intended as a proof-of-concept demonstration of the proposed IMU–RGB-D fusion and decomposition framework and, therefore, does not represent a general assessment of comfort, fatigue, or general postural strategies. Accordingly, the recordings used a single seat and mounting configuration to validate the fusion and decomposition layer under realistic service conditions, rather than to benchmark variability across seats, postures, or vehicle suspensions. Several sessions of 5–15 min were collected along segments with straight track, curves, acceleration, and braking.

The IMU—RGB-D fusion framework was instantiated using three inertial measurement units (IMUs) and one RGB-D camera on a common time base. A seat IMU was rigidly attached to the seat frame to represent the seat and vehicle deck vibration. A body IMU was mounted on the chest to capture the biodynamic response of the upper body. A camera IMU integrated in the Intel RealSense D457 provided camera acceleration, angular velocity, and orientation quaternion, enabling quantification and later compensation of visual sensor jitter. The RGB-D camera, mounted on the interior structure via a window suction mount, operated at video rate and was used to extract 3D landmarks of the nose and both shoulders as inputs to translational postural compensation metrics.

**Figure 2:**
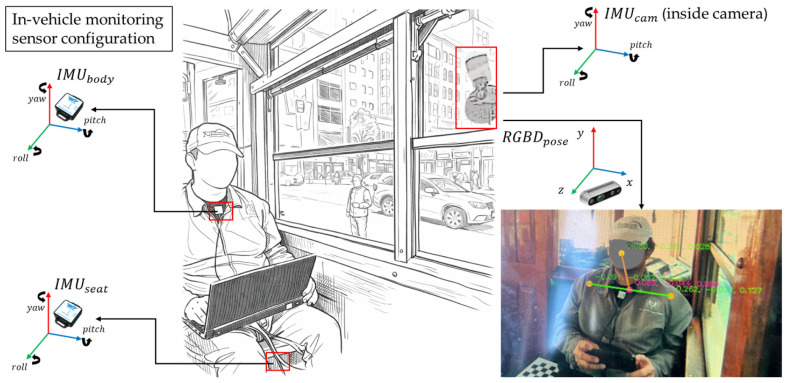
The experimental setup for the in-vehicle monitoring fusion sensor configuration.

All sensors were connected to a processing computer in the cabin. IMU packets, which are acceleration, angular velocity, and quaternion, as well as RGB-D frames, including colour and depth, were read continuously, time-stamped with the system clock, and stored as raw time series. The camera intrinsic parameter was also generated for calibration. Prior to recording, seat and body IMUs were initialized at zero point in a static upright posture, and an extrinsic calibration using a checkerboard was performed to relate camera and world coordinates. During acquisition, 2D landmarks from MediaPipe Pose and aligned depth values were combined to reconstruct 3D landmarks in the camera frame. Also, the orientation of motion-compensated landmarks and full world-frame transformation were performed offline in the analysis pipeline. The overall experimental setup, including the instrumented seat, body-mounted IMU, and window-mounted RGB-D camera, is illustrated in Figure 2.

### 4.2. Signal Quality and Preprocessing

Before computing the main metrics, which are the Postural Compensation Index (PCI), vibration transfer ratio (VTR) and vibration velocity, the signals recorded synchronously under a single master clock were subjected to a quality control and preprocessing pipeline. The aims were to reject clearly invalid segments, enforce identical time indices across all modalities, and separate dynamic vibration components from quasistatic gravity and drift. A frame-level global mask was constructed on the unified timeline. If any channel of IMU or RGB-D violated its quality criteria, that frame was marked invalid and excluded from all subsequent analyses. Consequently, all reported metrics are computed on the same set of valid time samples, enabling consistent multimodal fusion and interpretation of seated body vibration independent of artefacts and camera jitter.

Each IMU provided tri-axial linear acceleration and angular velocity, while the camera IMU additionally yielded a quaternion from a Madgwick filter. An acceleration norm threshold of 30 m/s^2^ was used as a conservative gate to flag unrealistic spikes, which were then incorporated into the global mask. Gyroscope data were used directly for rotational PCI and implicitly validated through the same mask. Gravity was removed later by per-axis mean subtraction for RMS analysis and by 1–20 Hz band-pass filtering for vibration velocity computation.

**Figure 3:**
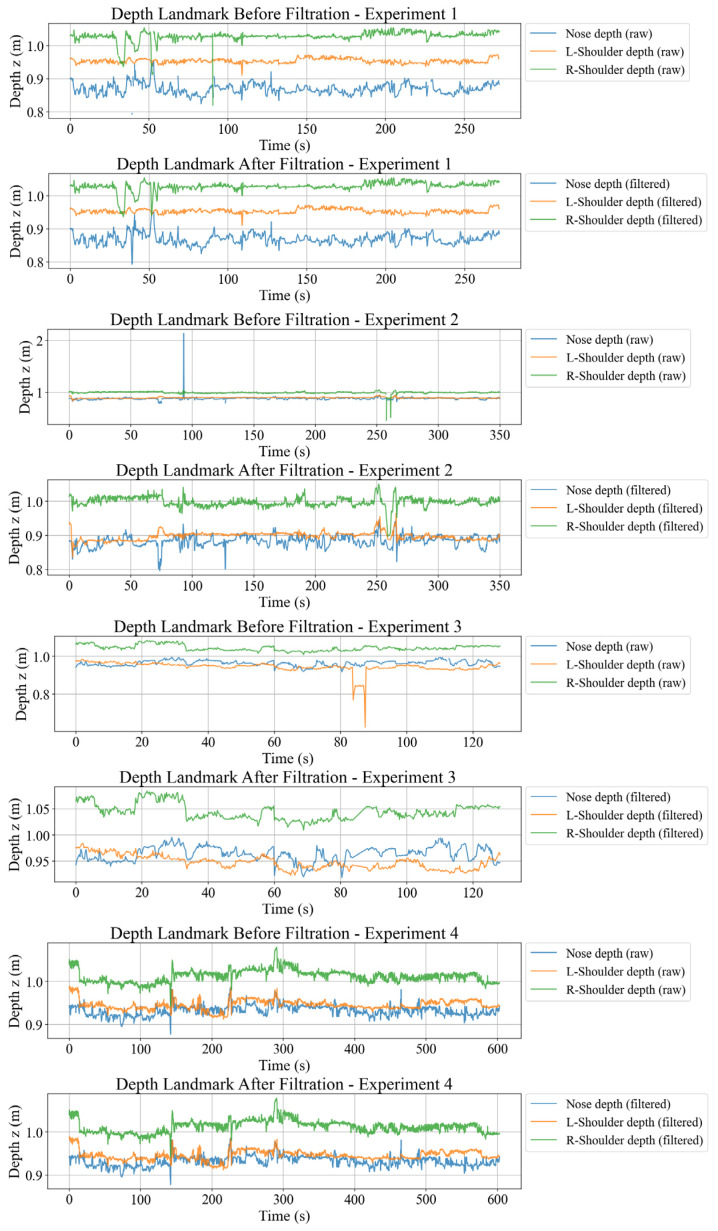
The visualization of the 4 experiments’ data quality control before and after filtration. The top panel shows the landmark depth time series in camera frames before filtration, showing the presence of depth spikes and short segments with missing values. The bottom panel shows the depth signal after filtration, where these problematic segments have been eliminated, resulting in a smoother and more consistent camera–body distance profile.

For the RGB-D stream, 2D landmarks from MediaPipe Pose and aligned depth values were combined to reconstruct 3D nose and shoulder points in the camera frame, then mapped to the world frame using a checkerboard-based extrinsic. Landmark level checks required all three points to be defined and within 0–2 m depth. Figure 3 shows how this filtering removes spikes and missing segments, yielding smoother depth trajectories.

After all quality criteria are applied, only the subset of frames that pass the global mask is used for the evaluation of PCI, VTR, and other metrics. Table 1 summarizes frame-retention statistics per session, indicating that most rejected frames stem from RGB-D limitations rather than IMU failures.

By applying cleaning at the unified frame level, any timestamp that is invalid in one channel is removed from all channels, so that the final multimodal dataset has consistent indices and has been normalized for quality. This curated dataset is then used to quantify seat-to-body vibration transmission, camera jitter and postural compensation patterns through rotational and translational PCI.

### 4.3. Seat to Body Vibration Transmission

**Figure 4:**
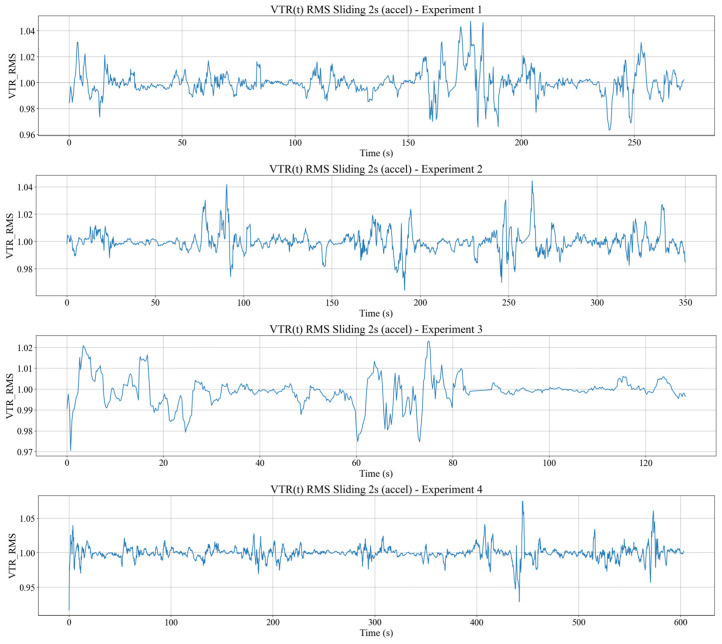
Chair-to-body vibration transmission ratio curve in 4 experimental data in the time domain (VTR(t)), which is defined as the ratio of the body’s calibrated acceleration magnitude to the seat at each time.

This section analyses how seat vibration is transmitted to the participant’s body in the time domain using three main metrics, which are the time domain vibration transmission ratio VTR(i), the RMS acceleration amplitudes of the seat and body computed over a 2 s sliding window, and the body vibration velocity obtained by spectral integration of bandpass filtered acceleration. All three metrics are evaluated on the same time segments so that the relationship between seat excitation and body response can be examined directly. The VTR(i) is defined as the ratio between the body and seat RMS acceleration magnitudes within each 2 s window, and visualized in Figure 4.

Figure 4 shows that VTR(i) value ≈ 1 indicates that the body experiences vibration levels comparable to the seat and behaves almost passively. Intervals where VTR(i) < 1 also appear when the tram crosses rail joints or during relatively mild disturbances, indicating that the part of the seat excitation is absorbed and damped by the seat–body system through passive soft-tissue deformation and micro postural adjustments. Transient peaks with VTR(i) > 1 are observed around specific events and can be interpreted as a combination of local resonance of body segments and active corrective movements.

**Figure 5:**
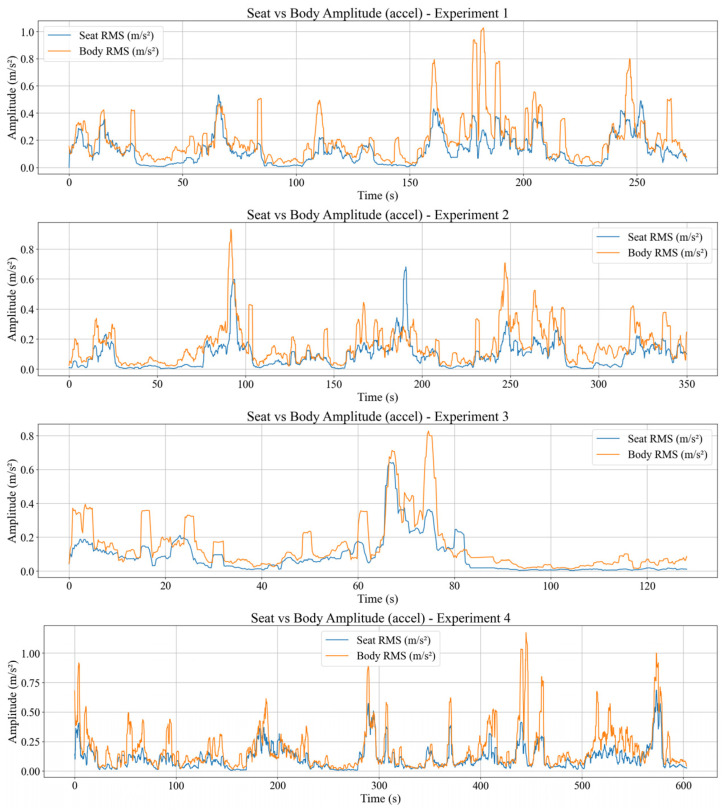
RMS acceleration amplitude of the seat and body in 4 experimental datasets, calculated over the same time window as the 2 s sliding window.

Aligning with that, Figure 5 shows the corresponding RMS acceleration amplitudes of the seat ($RMS_{seat}$) and body ($RMS_{body}$).

For most of the run, $RMS_{body}(i)$ follows $RMS_{seat}(i)$ with a small offset, consistent with $VTR\left(i\right)\approx 1$. Intervals with $RMS_{body}(i)\;<\;RMS_{seat}(i)$ yield VTR(i) < 1, meaning that there is attenuation, whereas $RMS_{body}(i)\;>\;RMS_{seat}(i)$ yields VTR(i) > 1, meaning that there is local amplification and corrective motion.

**Figure 6:**
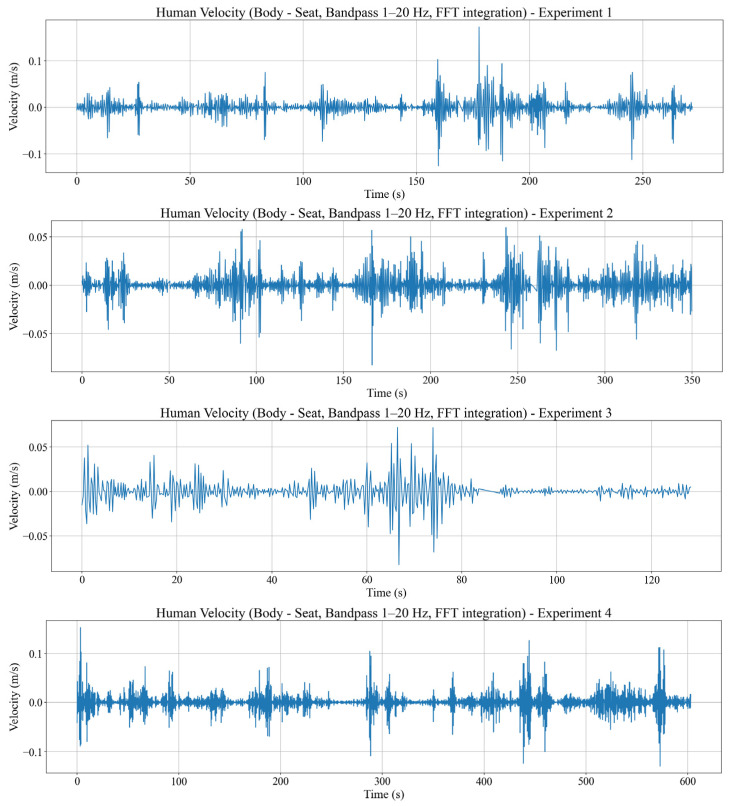
Body vibration velocity profile in four experimental data obtained from the seat and body differential acceleration signals.

In addition, body vibration velocity v(t) is obtained by forming a differential acceleration signal, applying a 1–20 Hz bandpass filter, and integrating in the frequency domain while suppressing components below 0.8 Hz. The body vibration velocity calculation results from four experimental datasets can be seen in Figure 6.

The resulting v(t) remains at moderate levels for most of the route, with clear increases at rail joints, curves, and during acceleration and braking. Within the proposed framework, v(t) serves as a bridge between acceleration-based transmission metrics VTR and fatigue/comfort indicators and provides a promising feature for future pre-fatigue detection and operator monitoring in dynamic environments.

### 4.4. Stability of the Visual Sensing System

This section examines the stability of the visual sensing system, which is critical for maintaining reliable 3D body landmarks from the RGB-D camera and, hence, for interpreting translational body motion within the proposed framework. The camera module is mounted on the tram interior and is therefore subjected to vibration induced by the dynamic environment of the tram. An embedded camera IMU provides acceleration, angular velocity and orientation, allowing us to quantify seat-to-camera vibration transmission and to compensate camera orientation when reconstructing 3D landmarks.

**Figure 7:**
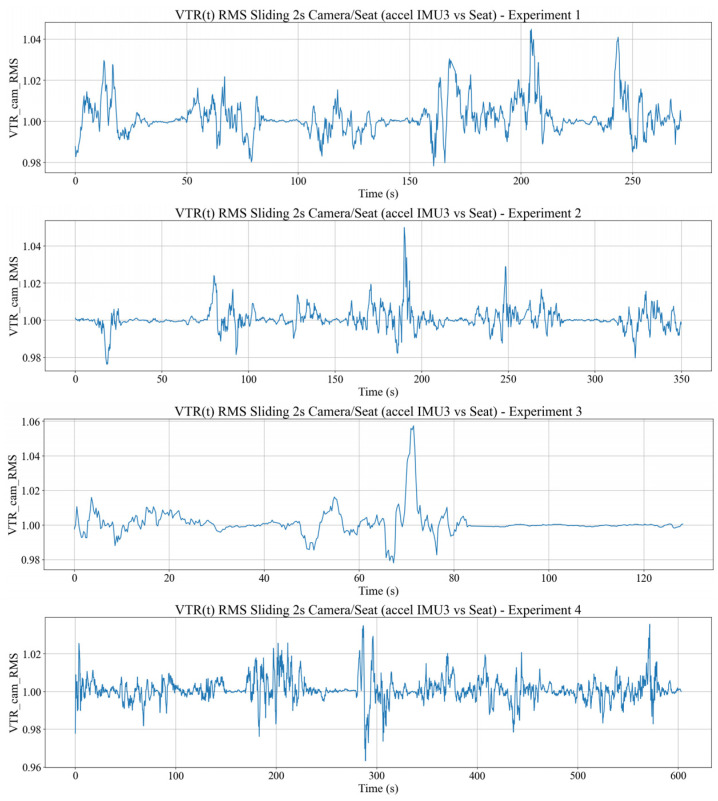
Vibration transfer ratio from seat to camera of four experimental data, calculated by performing 2 s sliding windows RMS on acceleration data.

The vibration transmission of the seat to the camera is characterized by a time domain vibration transmission ratio, $VTR_{cam}\left(i\right)$, computed as an acceleration RMS ratio over 2 s windows, analogous to the seat–body VTR. The $VTR_{cam}\left(i\right)$, over time, from the four experimental datasets, can be seen in Figure 7.

**Figure 8:**
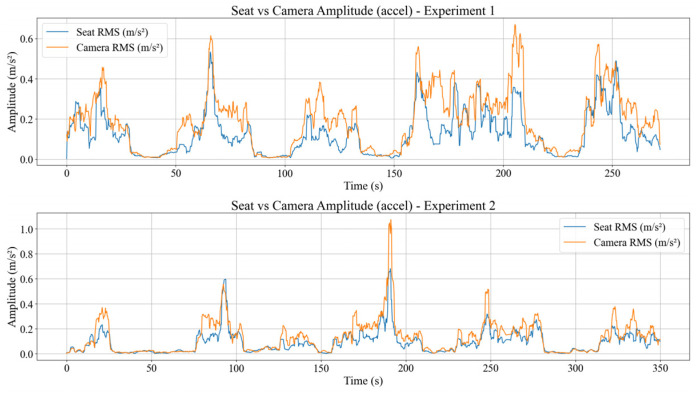

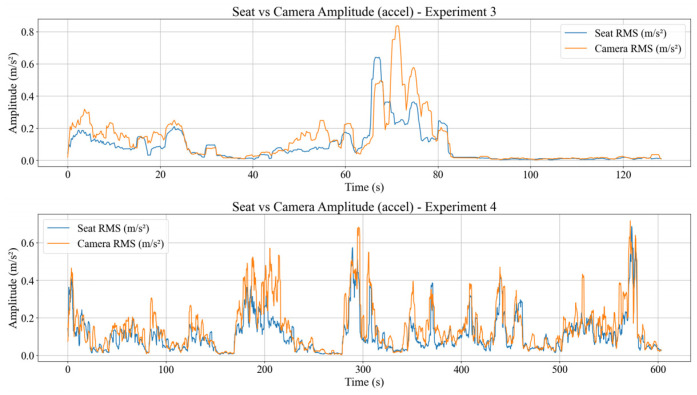
RMS acceleration amplitude of the seat and camera in four experimental data, calculated over the same time window as the 2 s sliding window.

Values $VTR_{cam}(i)\approx 1$ indicates that camera acceleration is comparable to seat acceleration, whereas values below or above one indicate relative attenuation or amplification. In the measured data, $VTR_{cam}(i)$ fluctuates around one and follows the excitation pattern of the seat, with transient peaks >1 during rail joints or sharp longitudinal acceleration changes. Figure 8 shows that camera RMS acceleration is typically slightly lower than, but strongly correlated with, seat RMS, confirming that camera jitter is primarily driven by tram vibration.

The $RMS_{seat}\left(i\right)$ curve represents the structural excitation level, while the $RMS_{cam}\left(i\right)$ curve indicates the jitter level experienced by the visual sensing system. In the observed data, the camera’s RMS amplitude is generally slightly lower than that of the chair but is highly temporally correlated. This indicates that the camera does not completely rigidly follow the chair, but rather dynamically experiences vibration patterns similar to those of the seat, so that the camera’s jitter is primarily driven by tram vibrations. The implication is that any fluctuation in $RMS_{seat}\left(i\right)$ is almost always followed by a fluctuation in $RMS_{cam}\left(i\right)$, so that the camera’s jitter peaks can be predicted from the dynamics of the chair’s vibrations.

**Figure 9:**
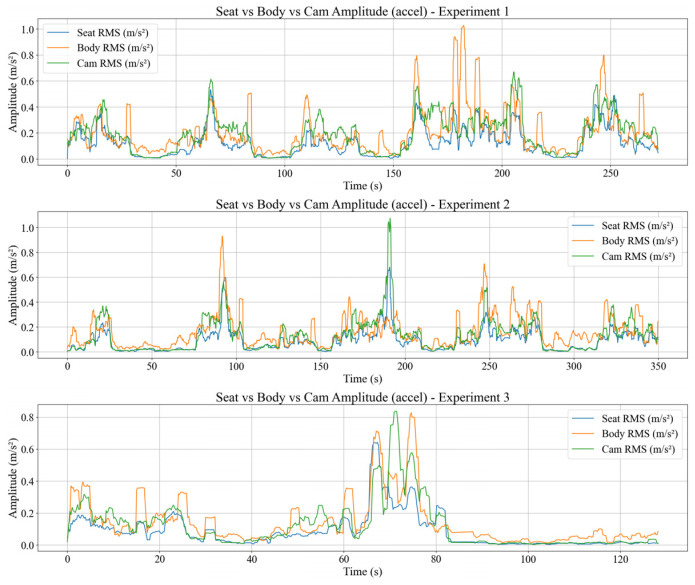

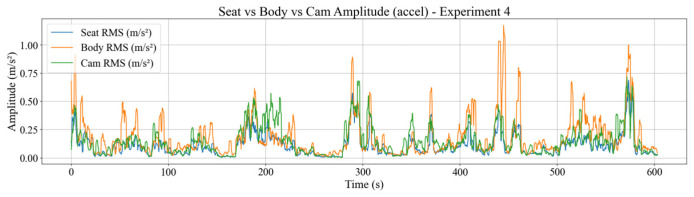
Time-domain vibration levels from the three IMUs (seat, body, and camera) across the four experimental sessions. The plot shows the sliding-window RMS acceleration magnitude RMS(t) (units: m/s^2^) computed using a ≈ 2 s window in the common world frame. Peaks coincide with dynamic events such as rail joints, curves, and acceleration/braking, highlighting that body RMS is generally highest while camera RMS can be comparable due to mount-induced jitter, motivating explicit camera section modelling.

To assess whether the camera is dynamically closer to the seat or to the body, the RMS amplitudes of the accelerations from all three IMUs are summarized in one compact representation. Figure 9 displays the RMS dynamics of the seat, body, and camera accelerations calculated from the median magnitude signal calibrated against the seat IMU, accompanied by each IMU’s RMS acceleration mean, as shown in Table 2.

Figure 9 and the accompanying Table 2 compare the mean RMS accelerations measured by the seat, body, and camera IMUs. Overall, the body exhibits the highest mean RMS level, followed by the camera and the seat. Despite having a lower global average than the body, the camera still shows elevated RMS levels and pronounced transient peaks, consistent with its mounting on a flexible window structure that can behave like a cantilever and amplify local vibration modes. This indicates that the camera dynamically follows the seat motion but can exhibit locally larger amplitudes under specific excitation conditions, making it a sensitive probe for visual vibration. Consequently, camera-induced jitter is treated as a separate sensing layer and compensated using camera IMU orientation to prevent contamination of RGB-D landmark coordinates used in translational PCI and postural analysis.

### 4.5. Postural Compensation Index

This section examines how the body responds to seat excitation through postural adjustment, in both rotational and translational components. As defined in the methodology, the PCI is computed as a sliding-window RMS of the relative rotational motion between body and seat or the landmark translational motion relative to a neutral reference. In this proof-of-concept study, PCI is interpreted as a physically grounded proxy of relative postural adjustment under excitation. However, it may reflect overlapping contributions from passive body–seat mechanics and active neuromuscular control and therefore should not be treated as a direct measure of physiological activation. Thus, in this dataset, PCI indicates how strongly the observed posture deviates from the rigid body following of the seat under vibration.

**Figure 10:**
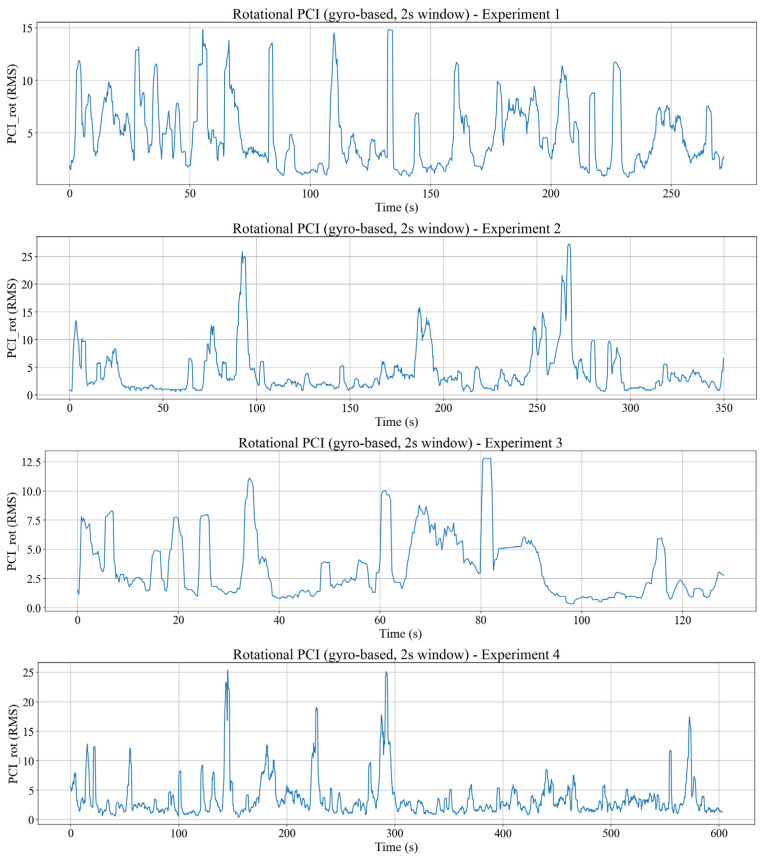
Rotational Postural Compensation Index ($PCI_{rot}$) across four experimental sessions. $PCI_{rot}$ is the sliding-window RMS of the relative angular velocity between body and seat IMUs (units: rad/s), computed over a ≈ 2 s window. Low values indicate near rigid body following of seat rotation, while transient peaks occur during acceleration/braking or sharper disturbances, reflecting increased relative torso rotation demands.

Rotational PCI denoted as $PCI_{rot}$ is derived from the difference between the angular velocities collected from the gyroscope of the body and seat IMUs over a 2 s sliding window. Low $PCI_{rot}$ values correspond to phases where the torso rotates almost in phase with the seat, whereas peaks in $PCI_{rot}$ indicate stronger relative angular motion of the torso with respect to the seat, consistent with increased postural adjustment demands during dynamic events. The human body rotational PCI results from four experimental datasets can be seen in Figure 10.

As shown in Figure 10, $PCI_{rot}$ remains at a low, stable baseline along relatively smooth track segments, suggesting near passive transmission of seat motion. Sharp increases in $PCI_{rot}$ appear around acceleration and braking events, coinciding with elevated RMS accelerations and increased body vibration velocity, consistent with increased angular adjustments to maintain orientation. In other intervals, $PCI_{rot}$ stays moderate despite higher seat RMS, suggesting that some phases may involve increased stiffness/bracing or passive absorption without large observable rotations, which cannot be uniquely separated without additional instrumentation. $PCI_{rot}$ therefore quantifies the degree of relative upper body on top of the transmitted vibration.

**Figure 11:**
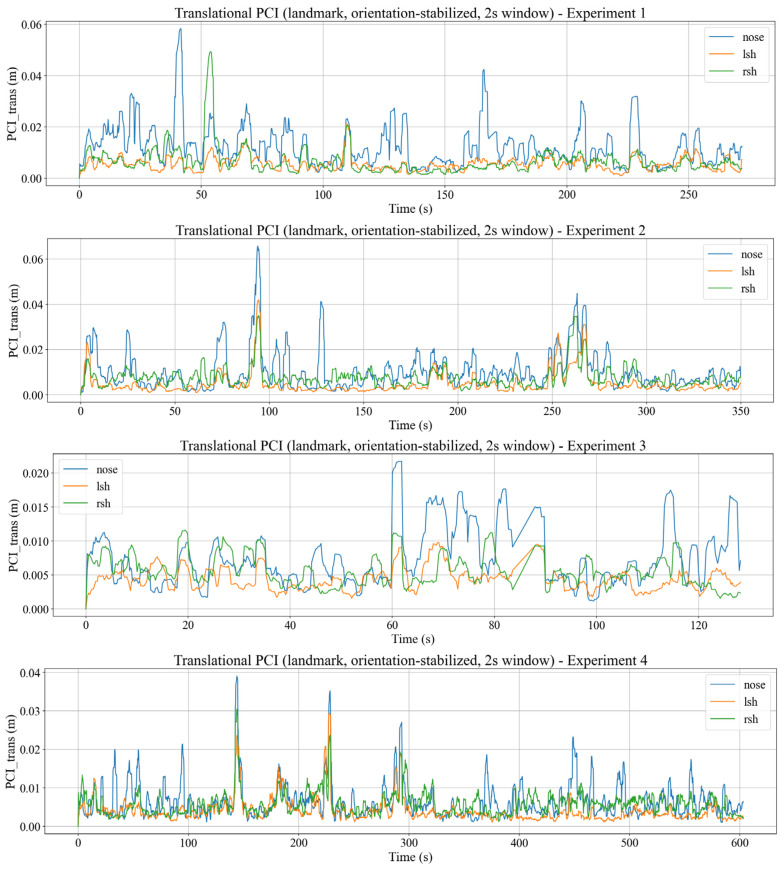
Translational Postural Compensation Index ($PCI_{trans}$) for nose and shoulders in four experimental sessions. $PCI_{trans}$ is computed as the sliding-window RMS of landmark translational velocity relative to a neutral reference (unit: m/s) in the world frame over a ≈ 2 s window, after camera jitter compensation. The nose typically shows larger values than the shoulders, indicating higher head motion relative to the upper torso and revealing segment-specific adjustment patterns.

Alongside $PCI_{rot}$, the translational PCI ($PCI_{trans}$) was also evaluated to obtain the human body translational impact of the vibration signal involving the RGB-D landmark. After orientation stabilization using the camera IMU quaternion and mapping to world coordinates, $PCI_{trans}$ is then computed for the nose $PCI_{nose}(t)$, left shoulder $PCI_{lsh}(t)$, and right shoulder $PCI_{rsh}(t)$ landmarks. Figure 11 shows $PCI_{nose}(t)$, $PCI_{lsh}(t)$, and $PCI_{rsh}(t)$ as the sliding RMS of RTVs in world space.

From that data, the $PCI_{nose}$ consistently exhibits the highest PCI values, consistent with the head acting as the most mobile segment and the end-effector of postural chains. The $PCI_{lsh}$, and $PCI_{rsh}$ show lower and more symmetric PCI, with occasional small asymmetries associated with mild lateral leaning. Temporally, peaks in PCI_trans often occur near, but not always at, peaks in VTR(t). This pattern indicates phases in which increased seat–body transmission does not necessarily trigger large translational compensation, as well as phases in which posture appears to be adjusted to support head and upper-body stability. Even under moderate excitation, noting that subjective comfort is not directly assessed in this study.

When PCI is examined jointly with VTR(t) and body vibration velocity v(t), a richer picture of postural strategy emerges. VTR(t) quantifies how much excitation is transmitted from seat to body, vibration velocity v(t) reflects the energy level of the resulting body motion, and PCI, both rotational and translational, indicates how strongly the postural control system responds with compensatory movements, particularly in the head and shoulders. This combination provides a richer description of seat–body transmission and associated relative postural adjustments in dynamic vehicular environments. While subjective comfort or fatigue is not directly measured here, these indices can inform future work toward comfort-oriented analysis and pre-fatigue monitoring once validated on larger cohorts and with additional reference measurements.

### 4.6. Axis-Wise Vibration Characteristics and Posture Compensation Analysis

This section analyses the axis-wise characteristics of vibration response and postural compensation resulted by this proposed framework. While previous analyses considered scalar magnitudes, e.g., RMS and PCI norms, here the distribution of motion energy across axes is examined. World coordinates are defined as lateral left–right (x), vertical (y), and anteroposterior (z) relative to the seat. Table 3 summarizes, for four experimental datasets, the percentage contribution of each axis to the dynamic body-IMU differential acceleration ${a_{body}}_{diff}(t)$ and to the translational PCI of the nose $PCI_{nose}(t)$, left shoulder $PCI_{lsh}(t)$ and right shoulder $PCI_{rsh}(t)$.

Across all experiments, the z component of ${a_{body}}_{diff}(t)$ consistently contributes the largest share of dynamic acceleration energy, with x and y contributing smaller and roughly comparable fractions. This indicates that the seated body’s vibration response is dominated by anteroposterior sway, which is biomechanically consistent with a passenger facing the direction of travel. It can also be said that changes in tram speed primarily induce forward–backward rocking against the backrest, while lateral and vertical components remain secondary.

Analogous with the ${a_{body}}_{diff}(t)$, $PCI_{trans}(t)$ exhibits distinct axis patterns for head and shoulders. For $PCI_{nose}(t)$, the lateral x axis is dominant in all sessions, followed by the anteroposterior z axis, while the vertical y contribution is always smallest. This suggests that head compensation is expressed mainly through left–right corrections, with moderate forward–backward motion and minimal vertical displacement, consistent with a route that imposes substantial lateral oscillations due to curves. In contrast, $PCI_{lsh}(t)$ and $PCI_{rsh}(t)$ are dominated by z, then x, with minimal y again, indicating that the chest and shoulders move primarily in the anteroposterior direction relative to the seat, consistent with rocking against the backrest during acceleration and braking, with lateral sway as a secondary component.

Overall, these axis-wise results show that seat-transmitted vibration leads to a highly anisotropic postural compensation pattern. The lower body and chest mainly respond to anteroposterior excitation, whereas the head plays a key role in lateral regulation. This directional context is important when interpreting PCI as an indicator of directional postural adjustment demands under excitation.

### 4.7. Anthropometric Consistency and Landmark Stability

**Figure 12:**
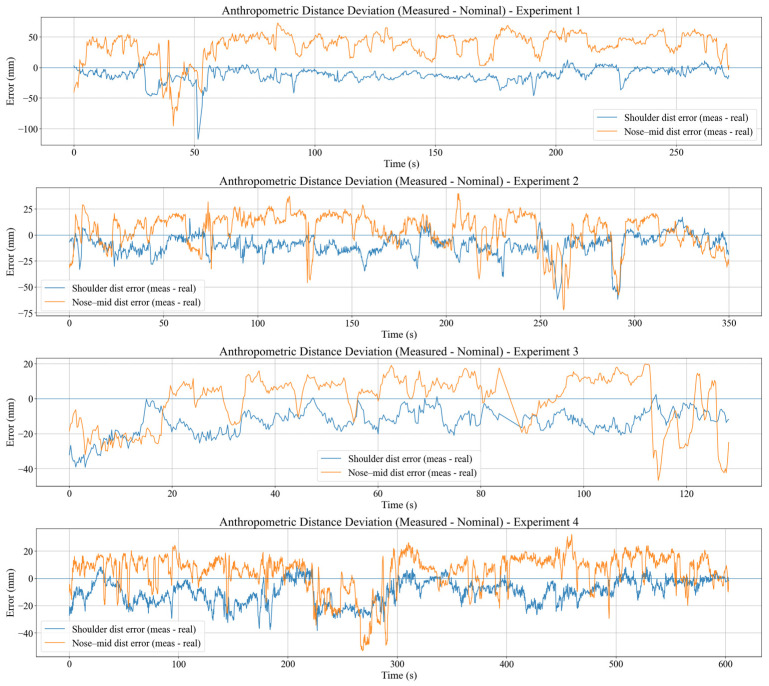
Anthropometric consistency of reconstructed 3D landmarks across four experimental sessions. The figure shows deviations of reconstructed shoulder width and nose–mid-shoulder distance from tape-measured nominal values (units: mm) after filtering and quality masking. Shoulder width deviations remain within a small band with minimal drift, supporting landmark stability for downstream PCI computation, whereas nose–mid-shoulder deviations are moderately larger due to genuine head posture changes and higher nose-landmark sensitivity.

Complementing the vibration and PCI analyses, this study also evaluates the quality of RGB-D–derived 3D landmarks from an anthropometric perspective. Two simple distances are used as indicators for evaluation, which are the shoulder width to measure the distance between left and right shoulders, and the nose–mid-shoulder distance to measure the distance between the nose and the midpoint of the shoulders. For the participant, both distances were first measured physically with a tape measure and recorded as nominal values. During the experiments, these nominal values were compared with distances reconstructed from MediaPipe-based skeletal landmarks using the RGB-D depth data, after applying the signal filtering and landmark motion compensation steps described earlier. The differences are expressed as anthropometric deviations in millimetres: $e_{sh}\left(t\right)=d_{sh_{meas}}\left(t\right)-d_{sh_{real}}$ for shoulder width and $e_{nm}\left(t\right)=d_{nm_{meas}}\left(t\right)-d_{nm_{real}}$ for nose–mid-shoulder distance. The measurement result was then plotted as functions of time in Figure 12. Prior to result interpretation, the MediaPipe-posed estimation model typically exhibits joint position errors on the order of several centimetres with respect to marker-based ground truth [86], so a realistic target for 3D anthropometric deviations in this setup is within approximately ±10–30 mm.

For shoulder width $e_{sh}\left(t\right)$, deviations across all four sessions remain within roughly ±10–20 mm of the nominal value. The bias is relatively stable along each route, with a slight tendency towards negative values in some sessions, likely reflecting a systematic bias in MediaPipe’s shoulder localisation for a seated subject. Occasional larger spikes up to about 100 mm appear in the raw data, but most of these extreme outliers are eliminated by the quality filters. Figure 12 only shows frames that pass the filters, so the remaining range reflects normal estimation error rather than gross failures. No long-term drift is observed, and mean deviations remain close to a fixed offset of a few millimetres to 2 cm, indicating that camera–world extrinsic and coordinate transforms are stable. Shoulder width variation is therefore mainly driven by local skeleton jitter and small posture changes.

Meanwhile, nose–mid-shoulder deviations $e_{nm}\left(t\right)$ exhibit a somewhat wider spread. For most of the recording, errors lie within ±15–30 mm, but certain segments exceed 40 mm, particularly when the participant corrects head posture or when tram-induced jitter affects skeleton fitting. Two factors explain this behaviour. First, genuine head motion, such as neck flexion/extension and nodding, changes the geometric distance between nose and mid shoulder, so this distance is not a pure anthropometric constant but reflects both body geometry and instantaneous posture. Second, the nose landmark is more sensitive to camera jitter and detection noise because it is a small feature with high contrast. Small orientation errors or pixel-level noise can translate into relatively large depth shifts. This is consistent with the translational PCI results, where the nose shows higher translational activity, especially in lateral and anteroposterior directions.

## 5. Discussion

This study introduced a visual–inertial fusion framework utilizing IMU-RGB-D data for isolating seated human body vibration in dynamic environments and instantiated it in an in-service urban tram case study. The framework integrates three IMUs that are mounted on the seat, on the chest representing the upper torso, and inside the RGB-D camera module, with RGB-D-based tracking of nose and shoulder landmarks. All data captured are synchronized on a common time base and filtered through a global frame-level quality mask. Across the four recording sessions, between 96 and 98% of frames were retained after quality data control, with most dropped frames caused by RGB-D depth loss or spikes rather than IMU failures, indicating that inertial sensing remained stable while the depth channel was the dominant limitation. The results reported are based on a single healthy participant (a member of the research team) and are intended as a proof-of-concept demonstration of the proposed framework in a real in-service tram environment. Accordingly, the observed PCI/VTR patterns and axis-wise behaviours should not be interpreted as general biomechanical, ergonomic, comfort, or fatigue-related conclusions. Generalization will require validation across a larger cohort of independent participants, repeated sessions, and, where feasible, reference measurements.

### 5.1. Summary of Key Findings

On the inertial side, the framework decomposes vibration into seat, body, and camera components. Time domain seat–body vibration transmission, quantified by a 2 s sliding window $VTR\left(t\right)$, as displayed in Figure 4, typically fluctuates around unity along smoother track segments, indicating near passive transmission, but intermittently dips below one when the body effectively attenuates seat vibration and rises above one around specific events such as rail joints, curves, and pronounced acceleration or braking events. These patterns are consistent with the paired RMS analysis as shown in Figure 5, where intervals with ${RMS}_{body}\left(i\right)<{RMS}_{seat}(i)$ correspond to $VTR\left(i\right)<1$ and intervals with ${RMS}_{body}\left(i\right)>{RMS}_{seat}(i)$ to $VTR\left(i\right)>1$. The derived body vibration velocity v(t), computed from the differential body–seat acceleration using 1–20 bandpass filtering and spectral integration with suppression frequency below 0.8 Hz, as visualized in Figure 6, remains at moderate levels for most of the route but shows clear peaks at rail joints, curves, as well as during acceleration and while braking. This metric provides an interpretable measure of vibration intensity that complements VTR(t), linking seat–body transmission to an interpretable intensity-related quantity beyond solely acceleration data.

The framework also quantifies camera vibration and its relation to seat and body motion. The seat–camera transmission ratio ${VTR}_{cam}(t)$ displayed in Figure 7, and the corresponding RMS curves in Figure 7 show that camera jitter closely follows seat excitation, with values fluctuating around one and transient peaks during sharper disturbances. When all three IMUs are compared together as shown in Figure 9 and Table 2, the body exhibits the highest mean RMS level (≈0.722–0.885 m/s^2^), followed by the camera (≈0.699–0.807 m/s^2^) and the seat (≈0.485–0.553 m/s^2^). Thus, although the body experiences the largest overall vibration, the camera still shows comparable magnitudes with pronounced transient amplification, consistent with its mounting on a more flexible window structure that can locally amplify vibration modes. This justifies treating camera-induced jitter as a separate sensing component that must be compensated using camera IMU orientation when interpreting RGB-D landmarks in translational PCI and postural analysis.

Postural responses are captured by the PCI. Rotational PCI, ${PCI}_{rot}(t)$, derived from body–seat gyroscope differences as shown in Figure 10, stays low and stable over smoother segments, indicating near-passive transmission, but exhibits sharp peaks coincident with increased RMS and vibration velocity, reflecting angular corrections of the torso relative to the seat. Translational PCI metrics computed from motion-compensated world frame landmarks for the nose and shoulders, as visualized in Figure 11, further refine this picture. Across all sessions, the nose PCI exhibits the largest lateral translational component, while the left and right shoulder PCIs are lower in aggregate and more symmetric, with only mild asymmetries associated with occasional lateral leaning.

Axis-wise analysis of the differential body acceleration, as displayed in Table 3, shows that the anteroposterior component contributes roughly 37–43% of the dynamic energy, with lateral and vertical components sharing the remaining 57–63% in comparable proportions, indicating that torso vibration is dominated by forward–backward sway against the backrest. In contrast, the nose translational PCI is dominated by lateral motion, frequently exceeding 50–60% of the energy, while shoulder PCI is dominated by anteroposterior motion, often above 50–65%, with vertical contributions remaining comparatively small. This axis-wise pattern indicates that the chest and shoulders chiefly respond to anteroposterior disturbances, whereas the head plays a key role in lateral regulation.

Finally, anthropometric consistency metrics provide a sanity check on the geometric stability of the fused skeleton. As shown in Figure 12, after filtering and stabilizing the landmarks, reconstructed shoulder width deviations remain within approximately ±10–20 mm of the nominal measured tape value, with no detectable long-term drift, suggesting that the camera’s world extrinsics and world frame mapping remain stable. nose–mid-shoulder distance deviations are slightly larger, typically within ±15–30 mm but occasionally exceeding 40 mm during head posture changes or residual jitter, reflecting both the genuine variability of head position and the higher sensitivity of the nose landmark to depth and orientation errors. Together, these results show that the fused landmark trajectories are accurate enough to support PCI computation while still revealing the limits of skeleton precision in a vibrating tram environment.

Overall, the case study demonstrates that the proposed framework can decompose multimodal measurements into platform excitation, mechanically transmitted body vibration, active postural compensation, and camera-induced artefacts, while providing quantitative metrics and percentages that characterize each layer in a consistent way

### 5.2. Implications, Limitations, and Future Work

The quantitative findings have several implications for in-vehicle sensing and monitoring. First, the fact that only a few percent of frames are rejected and that almost all rejections stem from RGB-D depth issues rather than IMU spikes suggests that inertial channels can be treated as relatively reliable carriers of platform and body dynamics, while visual channels require careful quality control. In practical systems, this implies that IMUs can anchor the temporal structure of the analysis, with depth and skeleton information opportunistically fused when quality permits.

Second, the observed RMS hierarchy, which is seat 0.48–0.55 m/s^2^, body 0.72–0.86 m/s^2^, and camera 0.69–0.80 m/s^2^, confirms that camera mounts can behave like local amplifiers. Seat–camera VTR(t) values near or above unity mean that camera jitter is not a small perturbation but a major vibration layer that must be modelled and compensated. This is particularly relevant for any driver monitoring system that relies on 3D skeletons inside vibrating cabins. Without camera IMU based dejittering, skeleton trajectories will systematically inherit camera resonance effects, especially in directions where the mounting structure amplifies vibration.

Third, the axis-wise distributions and PCI patterns point toward anisotropic postural control strategies that cannot be captured by single scalar metrics. The dominance of anteroposterior components in torso vibration and shoulder PCI, compared with lateral dominance in head PCI, suggests that different segments specialize in handling different disturbance directions under seated conditions. This has implications for ergonomic design, such as backrest geometry or lateral support, and for fatigue indicators. A rise in lateral head PCI without corresponding changes in anteroposterior body PCI might signal a distinct type of instability compared with globally increased vibration.

Fourth, the anthropometric deviations provide a practical benchmark for acceptable skeleton quality in vibrating vehicles. Shoulder width deviations within ±10–20 mm and nose–mid-shoulder distance deviations mostly within ±15–30 mm indicate that, after filtering and doing motion compensation, landmark trajectories are sufficiently stable for metric computation, but they also set a realistic bound on the accuracy that can be expected from RGB-D pose estimation under such conditions. This deviation is substantially smaller than the several-centimetre landmark errors typically reported for MediaPipe-based skeletons, and it falls well below the magnitude of gross postural events that the system is intended to detect. At the same time, these ranges set a realistic bound on the accuracy that can be expected from RGB-D pose estimation under such conditions. Any downstream algorithm that uses these landmarks, such as for clinical gait surrogates or fine-grained postural diagnostics, must be designed with this noise envelope in mind.

At the same time, several limitations remain. While the present work demonstrates the proposed decomposition in fusion layer techniques, it should be regarded as a proof of concept rather than a population-level study. In particular, the PCI metrics used here are purely kinematic and are defined as relative motion between body and seat signals. As such, elevated PCI values are expected to coincide with periods of active neuromuscular control, but they may also include passive biomechanical resonance of the body–seat system, and cannot strictly disentangle these components. The experiments involve a single healthy participant, one tram configuration, a single torso IMU, and three upper-body landmarks, so generalization to different body types, postures, seat designs, and vehicle classes has not yet been established. The current pipeline also operates offline and does not explicitly address real-time constraints or long-term calibration drift. However, real-time in-vehicle deployment is feasible because the IMU-side processing, including filtering, alignment, and sliding-RMS metrics, is lightweight. The main computational bottleneck is RGB-D landmark extraction and depth association, which can be optimized via frame-rate decoupling (IMU vs. RGB-D), ROI downsampling, and multi-core CPU with optional GPU acceleration on an in-vehicle edge computer. In addition, the pose-estimation component is based on a generic RGB-D model that has not been specifically trained or optimized for in-vehicle depth imagery. Although bandpass filtering is used within specific steps of the pipeline, we do not provide an explicit quantitative benchmark against filtering-only baselines for supporting single modality filtering. Such benchmarking will be considered in future work under controlled settings.

Direct quantitative benchmarking against laboratory baselines, such as marker-based motion capture for posture ground truth or controlled WBV protocols, is also not feasible in the present in-service tram setting due to operational constraints and the inability to deploy reference instrumentation or enforce repeatable excitation conditions. Therefore, validation in this study is supported primarily through internal consistency and physical plausibility checks, including cross-sensor coherence of the seat/body/camera decomposition, temporal alignment of metric changes with acceleration and braking segments, physically interpretable axis-wise energy distributions, and anthropometric stability of reconstructed landmark distances after quality control.

The proposed framework is vehicle-agnostic in that it relies on synchronized multimodal sensing, a common world-frame representation, and a global quality mask, with decomposition and RMS-based metrics defined consistently across sessions. Vehicle-specific factors mainly concern sensor mounting geometry, camera intrinsics and extrinsic calibration in the new cabin, and the dominant excitation spectrum of the target vehicle and route. Therefore, transferring the method to cars, buses, trucks, or forklifts would require repeating camera calibration for the new geometry, ensuring rigid and repeatable IMU placements, and re-checking key parameters, such as depth validity thresholds and RMS window settings, against the sampling rate and expected vibration bandwidth in the new environment.

Operational conditions such as rail joint impacts and acceleration/deceleration phases were not explicitly logged in the present in-service tram recordings. In future deployments, we will include synchronized operational event logging and vehicle speed reference signals to enable explicit event-level plot annotations and condition interpretation, while the present study remains focused on establishing a physically grounded IMU–RGB-D decomposition framework. In particular, the fusion is used to support the exploration of how platform-induced motion propagates into both inertial measurements (seat/body/camera IMUs) and translational landmark trajectories, allowing camera/seat artefacts to be modelled rather than implicitly absorbed into body motion. Future studies will also consider validation on a larger and more diverse cohort (different body types), across seat and suspension configuration, as well as multiple vehicle classes with different operating conditions, to strengthen generalisability.

From a standards perspective, the present analysis focuses on derived metrics rather than formal ISO indices. Frequency-weighted RMS acceleration, Vibration Dose Value (VDV), Maximum Transient Vibration Value (MTVV), and reference biodynamic response functions, defined in AS 2670 [19] and ISO 5982 [27], are not computed explicitly in this study. Instead, the proposed framework yields decomposed signals that can provide natural inputs for these indices. Future work will therefore expand the participant pool and vehicle types, such as cars, buses, forklifts, trucks, and other rail vehicles, develop real-time implementations of the framework, and systematically map the proposed metrics to standardized comfort and health measures. The proposed time-varying VTR can be extended to frequency-domain transmissibility and compared against the ISO 5982 reference seat-to-head transmissibility (STHT), using the decomposed seat and body signals. Isolated body acceleration and the derived vibration velocity can be used to compute AS 2670 frequency-weighted RMS, VDV, and MTVV specific to the body, and to test whether velocity-based indices better correlate with discomfort and fatigue than acceleration alone. PCI can be used to segment ISO-based exposure measures into passive and active phases, enabling analyses between VDV during high-PCI intervals and VDV during low-PCI intervals, and potentially supporting new combined indices of vibration-related fatigue and STHT characterization. Axis-wise body and landmark activity can be combined with ISO axis-specific weighting filters to identify which directional components dominate standard comfort indices, and to examine whether anisotropic PCI patterns predict directional exceedances of ISO limits. Anthropometric deviations can serve as a skeleton-quality indicator, which means that the ISO exposure metrics would be computed only on intervals where shoulder and nose–mid-shoulder distances stay within acceptable bounds, ensuring that body kinematics used for comfort and health assessment are not corrupted by depth artefacts or camera jitter. The metrics used in this proposed framework are not meant to replace ISO indices, but to act as a decomposition front-end that provides cleaner, physically interpretable signals. The AS 2670/ISO 5982 metrics will be analyzed in further work to decompose seat and body signals, and examine how PCI and axis-wise patterns modulate standard exposure measures such as frequency-weighted RMS, VDV, MTVV, and seat-to-body transmissibility. Thus, that isolated body-vibration information can be directly linked to established guidelines on comfort, fatigue, and operational performance.

## 6. Conclusions

Bringing these findings together, the present study offers a structured contribution to in-vehicle vibration and posture monitoring, which can be summarized in three main points:

1. An IMU-RGB-D fusion framework that treats seat vibration, body vibration, postural compensation, and camera jitter as distinct but coupled components rather than assuming static platforms or cameras. The framework is built around a unified data representation and a global quality mask that ensures strict multimodal alignment.
2. The set of complementary vibration and posture metrics includes seat–body and seat–camera VTRs in the time domain, body vibration velocity derived from differential acceleration and spectral integration, rotational PCI from IMU gyroscope, translational PCI from world frame landmark trajectories, axis-wise anisotropy measures, and anthropometric consistency metrics for shoulder width and nose–mid-shoulder distance. Together, these metrics provide a rich description of how vehicle vibration is transmitted, modulated, and compensated by the seated body and by the visual sensing system.
3. A case study in a real tram environment demonstrates that the framework can be implemented in a realistic dynamic vehicle, without assuming static sensors or controlled laboratory conditions. The pipeline is modular with respect to vehicle type and sensor placement and can be adapted to other cabin vehicles, such as cars, buses, forklifts, and trucks, for applications in ergonomics, driver pre-fatigue monitoring, and in-vehicle human state assessment.

In conclusion, the proposed IMU-RGB-D fusion framework provides a structured way to interpret multimodal measurements of seated humans in dynamic vehicular environments. By explicitly treating seat vibration, body vibration, postural compensation, and camera jitter as distinct but coupled layers, and by grounding this decomposition in quantitative metrics and percentage-based summaries, the framework bridges WBV research, in-vehicle ergonomics, and visual–inertial fusion. The tram case study demonstrates that these ideas can be realized in a real vehicle and can reveal not only when and how strongly the body is excited, but also how it compensates across different directions and segments, and how visual sensing is affected by its own vibration layer. These results provide a methodological foundation for more nuanced and physically grounded monitoring of human state in intelligent transportation systems. A clear path is also opened for future work in which the decomposed seat and body signals are used to compute AS 2670/ISO 5982 exposure indices, and in which PCI, axis-wise distributions, and anthropometric deviations are linked to standard comfort and fatigue measures, extending the framework from proof-of-concept decomposition toward clinically and ergonomically actionable metrics.

## Figures and Tables

**Table 1:** The summary of data quality control.

Scene	Total Frame	Kept	Dropped	Causalities		
				NaN/Depth Lost	Depth Spike	IMU Spike
1	1180	1151	29	25	4	0
2	1489	1442	47	29	18	0
3	530	507	23	6	17	0
4	2591	2512	79	78	0	1

**Table 2:** Acceleration mean RMS from seat, body, and camera.

Experiment	${\bar{RMS}}_{seat}$ (m/s^2^)	${\bar{RMS}}_{body}$ (m/s^2^)	${\bar{RMS}}_{cam}$ (m/s^2^)
1	0.516	0.776	0.807
2	0.553	0.863	0.720
3	0.485	0.722	0.699
4	0.553	0.885	0.778

**Table 3:** Axis-wise percentage contribution of the seated human body vibration in a dynamic environment.

Exp	${a_{body}}_{diff}$(*t*) (%)			$PCI_{nose}$(*t*) (%)			$PCI_{lsh}$(*t*) (%)			$PCI_{rsh}$(*t*) (%)		
	x	y	z	x	y	z	x	y	z	x	y	z
1	28.8	28.5	**42.8**	**52.6**	19.9	27.5	21.5	28.6	**49.9**	32.4	18.2	**49.4**
2	36.1	27.2	**36.7**	**62.5**	12.0	25.6	**42.3**	21.1	36.6	32.3	5.3	**62.4**
3	27.9	28.7	**43.4**	**43.2**	22.6	34.2	12.6	23.7	**63.7**	20.9	11.1	**67.9**
4	30.9	26.8	**42.4**	**52.2**	16.7	31.1	24.7	23.5	**51.8**	30.9	6.0	**63.1**

(Note: Bold texts indicate the largest value/dominant axis-wise percentage contribution (x/y/z) for each metric within the same experiment).

## Data Availability

The data presented in this study are available on request from the corresponding author. The data is not publicly available due to privacy concerns.
